# Synthetic Thermo-Responsive Terpolymers as Tunable Scaffolds for Cell Culture Applications

**DOI:** 10.3390/polym14204379

**Published:** 2022-10-17

**Authors:** Gaby D. Lizana-Vasquez, Luis F. Arrieta-Viana, Janet Mendez-Vega, Aldo Acevedo, Madeline Torres-Lugo

**Affiliations:** Department of Chemical Engineering, University of Puerto Rico-Mayagüez, Mayagüez 00682, Puerto Rico

**Keywords:** thermosensitive polymer, NiPAAm, hydrogel, scaffold, cell culture

## Abstract

The use of tailored synthetic hydrogels for in vitro tissue culture and biomanufacturing provides the advantage of mimicking the cell microenvironment without issues of batch-to-batch variability. To that end, this work focused on the design, characterization, and preliminary evaluation of thermo-responsive, transparent synthetic terpolymers based on N-isopropylacrylamide, vinylphenylboronic acid, and polyethylene glycol for cell manufacturing and in vitro culture applications. Polymer physical properties were characterized by FT-IR, ^1^H-NMR, DLS, rheology, and thermal-gravimetric analysis. Tested combinations provided polymers with a lower critical solution temperature (LCST) between 30 and 45 °C. Terpolymer elastic/shear modulus varied between 0.3 and 19.1 kPa at 37 °C. Cellular characterization indicated low cell cytotoxicity on NIH-3T3. Experiments with the ovarian cancer model SKOV-3 and Jurkat T cells showed the terpolymers’ capacity for cell encapsulation without interfering with staining or imaging protocols. In addition, cell growth and high levels of pluripotency demonstrated the capability of terpolymer to culture iPSCs. Characterization results confirmed a promising use of terpolymers as a tunable scaffold for cell culture applications.

## 1. Introduction

In vitro cell culture is a well-defined tool that provides the advantage of studying specific cell and tissue behavior outside the in vivo setting. Numerous strategies have been developed for the culture of cells using in vitro 2D and 3D platforms to mimic the natural interactions between cells and their microenvironment [1,2]. However, one of the main challenges is to provide the appropriate mechanical, chemical, and biological cues to guide cell response, proliferation, and differentiation through these materials [3]. In addition, if cell encapsulation is required, there may be challenges in harvesting cells as their harvesting may require chemical or mechanical manipulation. These manipulations may potentially affect the cells.

Furthermore, cell therapies have demonstrated great promise in the treatment of chronic diseases. Their large-scale manufacturing process requires the development of high-throughput platforms to reduce cost and improve accessibility [4,5]. To overcome these challenges, the use of synthetic materials, such as cell culture scaffolds, could provide an alternative, due to their versatility in tailoring physical, mechanical, and chemical properties. In addition, their synthetic nature could increase reproducibility and minimize issues with batch-to-batch variability when compared to natural-based materials [6].

Hydrogel-based polymers are excellent candidates for this application because their physical and chemical structures can be tailored. They also have the capability of absorbing large amounts of water, rendering them biocompatible [7]. In the past, synthetic hydrogel materials based on poly(acrylic acid) (PAA) [8,9], poly(ethylene glycol) (PEG) [10,11,12,13], poly(vinyl alcohol) (PVA) [14,15,16], polyacrylamide (PAAm) [17], etc., have been used for multiple cell culture applications to improve scaffold properties. For instance, the development of films [12], fibers [18,19,20], multilayers [21], membranes [17], 2D [16], and 3D cell culture [10,15,16] cell encapsulation, and multiscale porosity for cell applications [11,12] have been investigated for these purposes. 

On the other hand, hydrogels containing N-isopropylacrylamide (NiPAAm) [22,23,24], N-vinylpyrrolidone (NVP) [25], hydroxyethyl methacrylate (HEMA) [26], and engineered PEG-based polypeptides [27] have also been employed in 2D [25] and 3D cell culture [22], cell sheet engineering [23,24], contact lenses [26], and micro- and nano-patterns [27]. For the most part, those polymers have been used for their well-defined structure, degradability, porosity, sol–gel transition, and capacity to co-polymerize with other monomers to improve their final characteristic and ease large-scale production [28,29]. 

Although all these platforms have promising biocompatibility, a few have low mechanical stability due to their lack of stiffness, low viscosity, and elasticity modulus. In a few cases, the matrix may change its properties over time [30,31]. Others have adequate mechanical properties but lack bioactive motifs necessary to promote the morphogenesis of cell proliferation [17,31]. Furthermore, others incorporate a combination of different fabrication techniques including, for example, multi-layered cultures combined with micro-patterning surfaces [32], multiple substructures [33], and multi-solvent systems [34]. Their practical application in a cell manufacturing setting may be complex. Thus, the control of the aforementioned parameters is critical for the success of synthetic scaffolds in cell manufacturing applications [35].

To overcome these challenges, we have molecularly designed a synthetic polymer that could be used to grow and test cells. The design criteria were to create a non-cytotoxic smart platform capable of encapsulating cells during culture, while providing the opportunity to easily remove them without significant mechanical manipulation. Furthermore, the platform should provide transparency to monitor cell growth by microscopy, the capability to easily incorporate molecular cues, and be affordable and reproducible. For this purpose, three main components were selected: poly(N-isopropylacrylamide), vinyl phenylboronic acid (VPBA), and polyethylene glycol monomethyl ether mono methacrylate (PEGMMA).

Poly(N-isopropylacrylamide), also known as pNiPAAm, was selected for its thermo-responsive capability close to body temperature. pNiPAAm experiences a Lower Critical Solution Temperature (LCST) in water at 32 °C. This property can be adjusted by copolymerization with hydrophobic or hydrophilic monomers [36,37,38,39]. To be able to easily incorporate biological cues or reversibly attach cells to the matrix, VPBA was incorporated. VPBA is a hydrophobic monomer that behaves like a Lewis acid and interacts with Lewis bases to form reversible covalent bonds [40,41,42]. Hence, it can accept protons of diol groups localized on glycoproteins of cell membranes. This allows the rapid formation of agglomerates and adhesion on the surface matrix by reversible covalent linkage. Therefore, this component can easily be used to attach biological cues, such as peptides, proteins, or growth factors, without performing complex chemistry. Although promising, the dual combination of these monomers still lacks the capability to monitor cells through microscopy since copolymers of NiPAAm and VPBA are completely opaque at 37 °C. To this end, PEGMMA was incorporated. This macromonomer possesses numerous chemical and physical properties, such as biocompatibility, incredibly high hydrophilic affinity, and bulkiness, that would improve transparency [43,44,45,46]. Therefore, we hypothesize that the polymerization of NiPAAm, 4-VPBA, and PEGMMA monomers could successfully yield reproducible, transparent, and thermo-responsive terpolymers. These tailored materials could be used as a synthetic scaffold to monitor cell functionality through in vitro cell culture or manufacture. 

The use of the individual binary combination of these monomers for cell culture, as well as other combinations, has been reported [45,46,47,48,49,50,51]. For example, Shakya et al. synthesized an adjuvant scaffold of pNiPAAm-co-VPBA [51]. Similarly, Reddy et al. successfully grew NIH-3T3 cells in 2D hydrogel films and 3D cryogel scaffolds by mixing poly(NiPAAm-co-VPBA-co-DMAEMA) and PVA [50]. Likewise, to promote cell attachment and subsequent detachment, Konishi et al. formed annealed nanofibers based on a random crosslinked copolymer of NiPAAm with butyl acrylate (BA) and N-(2-hydroxyethyl) acrylamide (HEAAm) [48]. On the other hand, the selected monomers have been previously used as binary combinations for different applications, such as the fractional purification of polysaccharides [52] and the capture of glycoproteins and acidic/alkaline proteins [53].

The aforementioned materials were characterized using several techniques to corroborate their physicochemical properties, including FT-IR, ^1^H-NMR, DLS, and rheological analysis. In addition, the ovarian cancer cell line SKOV-3, Leukemia Jurkat T cells, iPSCs, and the NIH-3T3 Fibroblast cell line were used to provide an assessment of the biological interaction of these polymers with mammalian cells.

## 2. Materials and Methods

### 2.1. Materials

For the polymerization reaction, reactants such as N-isopropylacrylamide (NiPAAm), 4-vinylphenylboronic acid (4-VPBA), and polyethylene glycol monomethyl ether monomethacrylate (PEGMMA) of two molecular weights (400 and 1000 g/mol) were used as monomers. Anhydrous ethanol, 2,2′-azobis(2-methylpropionitrile) (AIBN), and petroleum ether were used as the solvent, initiator, and precipitant, respectively. All reactants were used as received from the manufacturer (Sigma Aldrich, St. Louis, MO, USA) except PEGMMA (Polysciences, Inc. Valley Rd, Warrington, PA, USA). Industrial nitrogen gas was employed for the inert atmosphere (Praxair, Guaynabo, Puerto Rico). Aluminum dishes of 42 mL (Fisherbrand, Pittsburgh, PA, USA) were used to dry samples in the oven. Deuterated methyl sulfoxide ampules with 0.3% TMS from Acros Organic Company (Thermo Fisher Scientific, Waltham, MA, USA) were obtained for ^1^H-NMR analysis. Millex-GP syringe filter (Millipore Sigma, St. Louis, MO, USA) of 0.22 µm pore size and 33 mm diameter polyether sulfone (PES) membrane was used to sterilize the terpolymers samples. Dulbecco’s Modified Eagle’s Medium (DMEM), sodium bicarbonate, and penicillin-streptomycin solution (Sigma Aldrich, St. Louis, MO, USA) were used to culture NIH-3T3. Cell treated 25T (25 cm^2^), and 75T (75 cm^2^) flasks, phosphate fetal bovine serum (FBS) (Corning, New York, NY, USA), buffer solution (PBS), trypsin, and Ethylenediaminetetraacetic acid (EDTA) solution (Sigma Aldrich, St. Louis, MO, USA) were obtained to expand and culture NIH-3T3 and SKOV-3 cell lines. RPMI 1640 medium, sodium bicarbonate, and gentamicin solution (Sigma Aldrich, St. Louis, MO, USA) were used for the SKOV-3 and Jurkat T cell culture. 96-well plates (Corning, New York, NY, USA) were used to test the terpolymer with both these cell lines. iPSCs were expanded in 6-well plates (Corning, New York, NY, USA) with mTeSR1 (Stemcells Technologies Inc., Vancouver, BC, Canada) and replated with Versene (Thermo Fisher Scientific, New Jersey, NJ, USA). Accutase (Innovative Cell Technologies, San Diego, CA, USA) was used for cell dissociation. Fructose (Sigma Aldrich, St. Louis, MO, USA) was employed to facilitate cell harvesting from the terpolymer. For cell culture controls on iPSCs experiments, Matrigel (Corning, New York, NY, USA), Fibronectin (Sigma Aldrich, St. Louis, MO, USA), and Vitronectin xF (Nucleus Biologics, San Diego, CA, USA) were selected. Hoechst and Actin-red stain solutions (Thermo Fisher Scientific, Waltham, MA, USA), formaldehyde at 1% (*v*/*v*), methanol at 90% (*v*/*v*), and Triton X-100 at 1% (*v*/*v*) (Sigma Aldrich, St. Louis, MO, USA) were used in the staining experiments. Live/Dead Viability Kit (Invitrogen, Waltham, MA, USA) was obtained for cell viability and cytotoxicity. FlowBuffer-1 was prepared with 2.5 g of Bovine Serum Albumin (BSA) in 500 mL of PBS for antibodies analysis. FlowBuffer-2 was obtained from the mixture of 2.5 g of BSA and 50 mL of Triton X-100 at 1% in 500 mL of PBS. Trypan Blue staining solution (Sigma Aldrich, St. Louis, MO, USA) was employed for live and dead discrimination. Oct-4 from rabbit (Cell Signaling Technology, Danvers, MA, USA) and Alexa Fluor 488 from rabbit (Thermo Fisher Scientific, Waltham, MA, USA) were selected for pluripotency studies in flow cytometry.

### 2.2. Experimental Design

**Figure 1:**
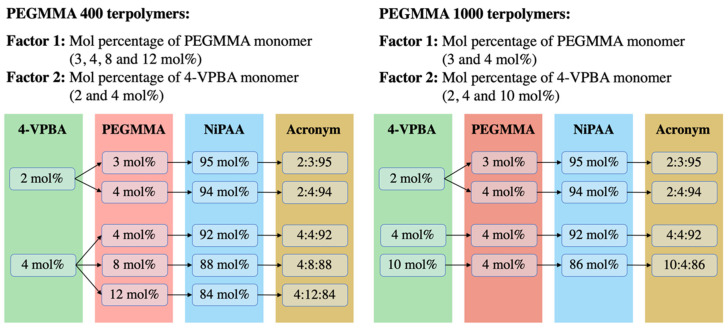
Factors and levels considered in 4-VPBA:PEGMMA:NiPAAm terpolymers experimental design.

One of the goals of this work was to find a monomer combination that could provide a transition temperature of around 37 °C. To achieve this goal, a balance between the hydrophobic and hydrophilic characteristics of VPBA and PEGMMA monomers is needed. These parameters were considered in the experimental design, along with the PEGMMA macromonomer molecular weight, since differences in its repetitive unit sizes may influence the transparency of the hydrogel. To this end, nine VPBA/PEGMMA/NiPAAm terpolymer combinations were obtained from the experimental design using two PEGMMA molecular weights (400 and 1000 g/mol). As can be appreciated in Figure 1, the same monomer combinations, 2:3:95, 2:4:94, and 4:4:92, were present for both PEGMMA molecular weights. Low and intermediate levels of 4-VPBA (2 and 4 mol%) and PEGMMA (3 and 4 mol%) were considered to control the LCST in the terpolymers. Furthermore, in the experimental design of the PEGMMA 400 terpolymers, the effect of molar composition (4, 8, and 12 mol%) on the transparency of terpolymers and thermal properties was investigated. Additionally, in the design of PEGMMA 1000 terpolymers, the high-level effect of 4-VPBA (10 mol%) was considered to balance the extra hydrophilicity behavior added by using a higher PEGMMA molecular weight. 

### 2.3. Terpolymer Synthesis

The methodology to polymerize p(VPBA-co-PEGMMA-co-NiPAAm) terpolymers was adapted from a previous study by Uguzdogan et al. in 2002 [54]. The reaction was a free radical polymerization on an inert atmosphere of nitrogen gas for 24 h at 65 °C, using AIBN and anhydrous ethanol (Sigma Aldrich, St. Louis, MO, USA) as a free radical initiator and solvent, respectively. The reaction was performed on an integral radial gas distribution system of six reactors (Radleys Carousel 6 Plus Reaction Station, Heidolph™, Wood Dale, IL, USA). 

For all desired compositions, detailed in Figure 1, the amount of initiator was 1.2% of the NiPAAm moles used. A total of 50 mL of anhydrous ethanol was employed for each reactor. From this volume, 5 mL was set aside to dissolve the initiator, and the remaining 45 mL was used to solubilize the monomers. Stock solutions of monomers/solvent and initiator/solvent were prepared and sonicated for 20 min in cold water to increase the solubility of monomers (Branson Ultrasonics Corp, Danbury, CT, USA). From the monomers/solvent stock solution, 50 mL was transferred to each reactor along with a magnetic stirrer. 

Reactors were placed in the reaction system and purged with nitrogen gas for 20 min, with a corresponding venting of pressure in the small neck of reactors, using a syringe needle. Nitrogen flow was maintained during the entire reaction time in the closed system. The solution of monomers was allowed to reach a constant temperature of 65 °C along with continuous stirring at 150 rpm, which was maintained throughout the entire reaction time. From the initiator/solvent stock solution, 5 mL was added to each reactor via a septum port using a glass syringe and needle to start the reaction. After 24 h of polymerization under the reaction conditions, the system was turned off, and the reactors were allowed to cool to room temperature. 

The content of each reactor was individually transferred to a 200 mL beaker to evaporate the solvent on a fume hood. Then, the terpolymer solution from each reactor was precipitated with 30 mL of petroleum ether, mixed for a few minutes, and the supernatant fluid was removed using a glass Pasteur pipette. The precipitation process was performed three times before transferring the sample to a 42 mL aluminum plate and placed in a vacuum oven (VWR1430, VWR, Radnor, PA, USA) at 50 °C for 48 h or until complete liquid evaporation. The terpolymer was recovered, crushed in a porcelain mortar and pestle to obtain a powder, transferred into a labeled glass vial, weighed, and stored for later characterization. 

### 2.4. Polymer Characterization

#### 2.4.1. FT-IR

Fourier Transform Infrared Spectroscopy was performed to confirm the success of the polymerization by the identification of principal functional groups present in the synthesized terpolymers. A dried terpolymer powder sample was placed on a SeZn ATR crystal covering all visible crystal area installed on a Nicolet iS50 FT-IR (Thermo Fisher Scientific, Waltham, MA, USA). Sample runs were 32 scans at a wavelength range of 4000 to 400 cm^−1^. In addition, a background scan, which was subtracted by the equipment by default, was performed before each analysis. The complete process was performed three times on all reactor samples for each terpolymer combination. 

#### 2.4.2. ^1^H-NMR

Proton nuclear magnetic resonance was used to confirm the success of the polymerization reaction and determine the mol% of each monomer present in the polymer chain. A polymer sample of 75 mg was dissolved in a 0.75 mL solvent ampule of deuterated methyl sulfoxide with 0.3% TMS. The analysis was performed in a 500 MHz UltraShieldTM nuclear magnetic resonance (NMR) spectrometer (Bruker, Billerica, MA, USA).

##### Molar Composition (mol%)

After synthesis, the mol% composition of each terpolymer combination was calculated with further analysis of ^1^H-NMR spectra using MestReNova Software. The mol% by ^1^H-NMR was calculated using the integral area under the peaks of the most important protons present in the terminals of each monomer, 2 (CH_3_) terminals of NiPAAm, 2 (CH_2_) of repetitive units of PEG, and 4 (CH) of ring terminal of VPBA, normalized with the total number of protons present on each peak. 

##### Number Average Molecular Weight (*M_n_*)

The end-groups analysis of ^1^H-NMR spectra allows calculating the average number of all molecule weights present in the polymer chain (*M_n_*). The corresponding resonance signal to protons in terminal groups is proportional to the species concentration. It can be compared with those signals of the chain repeating units, whose number is called the degree of polymerization (DP) [55,56,57]. Herein, the DP can be calculated by Equation (1):

(1) $$a_{i}=kn_{i}m_{i}\;\to \;k=\frac{a_{i}}{n_{i}m_{i}}$$

where *a_i_* is the ^1^H-NMR peak area or intensity of species *i, n_i_* is the number of species repeating units (DP), *m_i_* is the number of protons in the peak, and *k* is a constant. 

Rearranging Equation 1 and the constants (*k_v_ = k_p_ = k_n_*) of three moieties present in the terpolymer structure: *v* (4-VPBA), *p* (PEGMMA), and *n* (NiPAAm), gives Equation (2):

(2) $$\frac{a_{v}}{n_{v}m_{v}}=\frac{a_{p}}{n_{p}m_{p}}=\frac{a_{n}}{n_{n}m_{n}}\;\to \;n_{v}=\frac{a_{v}n_{p}m_{p}}{a_{p}m_{v}}\;and\;n_{n}=\frac{a_{n}n_{p}m_{p}}{a_{p}m_{n}}$$

Based on one PEGMMA molecule, which is known to have an average of 9 or 23 repetitive units in 400 and 1000 molecular weights, respectively, the PEGMMA repetitive unit (DP = *n_i_*) in the terpolymer was calculated. The DP of PEGMMA allows the proportional calculation of 4-VPBA and NiPAAm DPs. Finally, the *M_n_* (g/mol) was calculated by the sum of all moieties’ molecular weight present in the terpolymer structure multiplied by their calculated DP as described in Equation (3).

(3) $$M_{n}=\sum^{\;}functional\;groups\;M_{w}\times n_{i}$$

#### 2.4.3. TGA

The thermal stability of terpolymer was investigated using a Labsys Evo TGA/STA-EGA thermal gravimetric analyzer (TGA) (Setaram, Austin, TX, USA), operating with nitrogen gas as purge at 40 mL/min. Previously dried pNiPAAm and terpolymer p(VPBA-co-PEGMMA-co-NiPAAm) samples were heated from 25 to 600 °C under a nitrogen atmosphere with a heating rate of 10 °C/min.

#### 2.4.4. DLS

Experiments were carried out by preparing a polymer solution of 0.1 wt.% in deionized (DI) water. The polymer solution was then transferred to a plastic cuvette. Measurements were performed using a NanoBrook Omni (Brookhaven Instruments, Holtsville, NY, USA) equipped with a laser wavelength of 636 nm and a temperature-controlled cell compartment to determine the effective diameter. Samples were allowed to equilibrate for 180 s before a reading was taken. The temperature varied from 25 to 60 °C in increments of 0.2 °C. The scattered light was detected at 90° with the integration of 120 s. The effective diameter at different temperatures was collected, and R program version 3.6.3 (R Foundation, R Core Team) was used to calculate the inflection point of the curve that represents the LCST of each polymer. The raw data collected for each of the curves fitted to an equation derived to calculate the inflection point of the curve, which corresponds to 50% of the sudden change in diameter on the curve.

#### 2.4.5. Rheology

Rheological characterization was performed on an Anton-Paar Physica MCR302 equipped with a Peltier temperature control attachment using stainless steel parallel plates (d = 25 mm). Homogenized samples were loaded into the rheometer at room temperature. The sample and fixture temperature were allowed to equilibrate to the starting temperature (usually 15 °C) for at least 5 min. After the temperature reached equilibrium, rheological tests were performed. In the constant-stress temperature-ramp (CSTR) viscosity test, the viscosity was measured at a constant stress of 0.1 Pa from 15 to 60 °C at a heating rate of 3 °C/min. The dynamic viscoelastic moduli were measured at a frequency of 0.5 Hz and a strain of 1%, while the temperature ramps were from 15 to either 45 or 60 °C at a heating rate of 3 °C/min.

The thermomechanical properties of the gel were evaluated using two different dynamic oscillatory shear tests. First, by frequency sweeps, and later a time dependence of the viscoelastic shear moduli at constant temperature and deformation (i.e., frequency and strain). Frequency sweeps were evaluated from 0.01 to 20 Hz at a constant strain of 1% to guarantee measurements in the linear viscoelastic regime at constant temperatures of either 37 or 45 °C. Constant temperatures correspond to normal human body temperature and a temperature above LCST for all samples, respectively.

The stability of the formed structure was evaluated by monitoring the viscoelastic moduli for 20 min at a constant frequency of 5 Hz at a strain of 2% at either 37 or 45 °C. A homogenized fresh sample was loaded at room temperature for these tests, and the sample and fixture were heated to the desired test temperature. Rheological tests were performed after the temperature reached equilibrium.

### 2.5. Cell Assays

#### 2.5.1. Polymer Washing

Once terpolymer characterization was performed, terpolymer combinations that experienced LCST close to body temperature and reached transparency were selected to be tested in cell culture after polymer washing. For this purpose, a terpolymer solution in cold DI water was prepared in a ratio of 5 mL of DI water for each gram of polymer. The mixture was allowed to rest at 4 °C until proper mixing was achieved. The terpolymer solution was washed by thermo-precipitation by exposing the solution to 50 °C until polymer precipitation. After the thermo-precipitation, the sample was dissolved in 5 mL cold DI water and dried at 50 °C for 48 h or until complete liquid evaporation. The resulting samples were recovered and crushed with a porcelain mortar to obtain a powder, which was transferred into a labeled glass vial, weighed, and stored for later characterization.

#### 2.5.2. Sterilization

After washing, the terpolymer samples were sterilized by filtration using a gamma sterile syringe with a hydrophilic PES membrane filter. Dried samples were weighed and dissolved in fresh and cold DI water to form a 40 wt.% solution. In a laminar flow hood, using a plastic sterilized syringe, 5 mL of DI water was flushed through the filter to eliminate the possibly remaining residues of the manufacturing process. After removing the residual liquid with air, the terpolymer solution was filtered and stored on a previously sterilized glass vial for future use in cell culture. 

#### 2.5.3. Cell Culture 

##### NIH-3T3 Cell Line

Mus musculus embryonic fibroblast NIH-3T3 cells (ATCC, Manassas, VA, USA) from passages 24 to 32 were cultured in T25 flasks with a seeding density of 5000 cells/cm^2^ in complete medium. Complete DMEM medium was prepared using 1% penicillin-streptomycin as an antibiotic, sodium bicarbonate, and supplemented with 10% FBS. Cells were then incubated at cell culture conditions (37 °C and 5% CO_2_). PBS and Trypsin-EDTA were used to passage the cells after 80% of confluency [58].

##### SKOV-3 Cell Line

The SKOV-3 ovarian cancer cell line, kindly provided by MD Anderson Cancer Center, was maintained as described in a previous report [59] and sub-cultured in T25 cell culture flasks. PBS and Trypsin-EDTA were used for cell detachment after reaching 90% confluency. The SKOV-3 cells in passages between 18 and 30 were employed for terpolymer experiments.

##### Jurkat T Cell line

Clone E6-1 Jurkat T cells (ATCC^®®^ TIB-152™), purchased from ATCC (Manassas, VA, USA), were maintained at cell culture conditions in RPMI 1640 medium supplemented with 10% FBS and 1% penicillin-streptomycin solution. Cells were sub-cultured every 2 to 3 days, keeping a cell density between 1 × 10^5^ and 1 × 10^6^ viable cells/mL. For terpolymer testing experiments, cell passages between 5 and 20 were employed.

##### Induced Pluripotent Stem Cells (iPSCs) Cell Line

Human iPSCs cell line (WTC11, provided by the University of Wisconsin, Madison, WI, USA) was used to test 2:4:94_P400 terpolymer in a monolayer. Cells were expanded in 6-well tissue culture plates coated with Matrigel. The maintenance medium was mTeSR1 serum-free based on DMEM/F12 containing bovine serum albumin [60]. Cells were sub-cultured from passage 50 at 70–80% confluency with Versene.

### 2.6. Terpolymer Testing

#### 2.6.1. Cell Encapsulation

Using a 40 wt.% sterilized terpolymer solution in DI water, different concentrations of terpolymer were prepared by dilution with 1X cell culture media to test the SKOV-3 and Jurkat cell lines in 96-well plates. Various ways of employing the terpolymer for cell culture were studied. Terpolymer scaffolds were studied as a 2D coating, and cells in suspension were encapsulated in terpolymer scaffolds using the sandwich and mixing conditions to form a 3D cell culture. To create the coating, a volume of 100 µL of terpolymer solution was added to each well and incubated at cell culture condition for 3 h. This volume is enough to cover the surface area of a flat-bottom 96-well plate with terpolymer gel. For the sandwich condition, an additional 50 µL terpolymer solution was resuspended with 50 µL culture media containing cells. Then, the suspended cells were gently seeded above a previously prepared polymer-coated plate, and incubated at cell culture conditions. For the mixing condition, 100 µL of cells in suspension were mixed with 100 µL of terpolymer solution. 

The SKOV-3 cell line was tested with 15, 20, and 25 wt.% terpolymer scaffolds using mixing and sandwich conditions, while Jurkat T cells were tested with 15 and 7.5 wt.% terpolymer scaffolds with all the conditions described above. In the case of a 7.5 wt.% scaffold, 200 µL of terpolymer solution was used to form the coating. In all experiments, an amount of 5000 SKOV-3 and 20,000 Jurkat cells were employed. The medium was removed and replaced with a fresh medium every other day to maintain the cells.

#### 2.6.2. Cell Harvesting

After the corresponding culture time, SKOV-3, Jurkat T, and iPSCs were harvested from the terpolymer scaffolds by taking the plates out of the incubator and allowing the temperature to drop to room temperature for at least 20 min. After the polymer changed to liquid form, cells were harvested (as individually described in the following sections) and manually counted. 

##### SKOV-3 Cell Line

Half of the total volume employed/well in a 96-well plate (100 µL) was removed and replaced with cold media at 4 °C (100 µL). A 20-min incubation was allowed to help the liquefication of terpolymer and facilitate cell harvesting. After incubation, a representative sample volume/well (150 µL) was transferred to a 1.5 mL microcentrifuge tube containing 350 µL of cold completed media (4 °C) to dilute the terpolymer content. Finally, cells were pelleted by centrifugation at 350 g for 5 min. A volume of 350 µL of supernatant was removed. Cells were resuspended with the remaining 150 µL. SKOV-3 spheroids were washed once with PBS and dissociated with 50 µL of Trypsin-EDTA. For this purpose, cells were incubated for 5 min with Trypsin-EDTA. The reaction was quenched with 100 µL of the completed cell medium (a total of 150 µL/sample). 

##### Jurkat T Cell Line

Similar to SKOV-3 cell harvesting, half of the total volume employed/well in a 96-well plate (150 µL) was removed and replaced with cold media (4 °C). A 20-min incubation was allowed to help the liquefication of terpolymer and facilitate cell harvesting. After incubation, a representative sample volume/well (200 µL) was transferred to a 1.5 mL microcentrifuge tube containing 350 µL of cold media (4 °C) to dilute the terpolymer content. Then, cells were pelleted by centrifugation at 350 *g* for 5 min. Finally, a volume of 350 µL of supernatant was removed, and cells were resuspended with the remaining 200 µL for further analysis.

##### iPSC Cell Line

In the case of iPSCs, cells cultured in a 24-well plate were exposed to 500 µL of fructose at 20 mM for 3 min. Then, fructose was removed, and cells were incubated with 500 µL Accutase for cell dissociation. In this step, the terpolymer was liquid. Lastly, cells were centrifuged at 200 rcf for 5 min to form a pellet and recover cells.

### 2.7. Cell Viability Assay

Cell viability assay of NIH-3T3 cells was performed with Live/Dead Viability Kit reagent as previously described [61]. In a 24-well plate, a number of 9500 cells suspended in regular medium was seeded per well. After 18 h of incubation, cell medium was removed, and cells were exposed to 700 µL of terpolymer solutions. Different concentrations (5, 10, 15, and 20 wt.%) of 2:4:94 P400 and 10:4:86 P1000 terpolymers were selected to verify cytotoxicity. After 24 h, the terpolymer solution in the wells was removed and washed twice with PBS. Regular cell culture medium was added to each well for 24 h to allow cells to recuperate before staining them with a Live/Dead Viability Kit. Cells were stained with calcein AM and ethidium homodimer-1. These stains can recognize viable or membrane-compromised cells by the binding of dye with nucleic acids. After 20 min of incubation, cells were recovered and transferred to 2 mL tubes for flow cytometer analysis using a BD Accuri C6 (BD Biosciences, NJ, USA) flow cytometer. 

Moreover, SKOV-3 and Jurkat T cells were manually counted using Trypan Blue staining solution for live and dead discrimination after cell harvesting from terpolymer scaffolds.

### 2.8. Cytoskeletal Staining 

Fixation and permeability of SKOV-3 and NIH-3T3 cell lines were performed using 4% Formaldehyde and 0.2% Triton solutions, respectively, before the staining step. Staining of the cytoskeleton (red) and nucleus (blue) of cells was performed using 100 µL of Actin Red (2 drops/mL) and Hoechst (1:1000 ratio) staining solutions, respectively [62]. All solutions were prepared with PBS, which was also used as the inter-reagent wash solution. 

### 2.9. Pluripotency Studies

iPSC cells were used as a model to test these polymers as potential synthetic substrates for iPSC culture. For this purpose, experiments were conducted to elucidate the capacity of cells to maintain pluripotency during 2D culture. Cells were harvested from the terpolymer coating by adding fructose and dissociated with Accutase. The expression of the octamer-binding transcription factor 4 (Oct-4), which is involved in the self-renewal of undifferentiated iPSCs, was tested by flow cytometry. Oct-4 from rabbits was selected to verify the pluripotency of cultured iPSCs. To observe Oct-4 expression by flow cytometry, Alexa Fluor 488 from rabbit was selected to bind with Oct-4. For the process, cells were fixed with paraformaldehyde and permeabilized with methanol at −20 °C overnight. Methanol on samples was removed by centrifugation and washed with FlowBuffer-1. Oct-4 antibody was added at a concentration of 1:400 to samples with FlowBuffer-2. Later, at 1 h, the excess of antibodies was removed by centrifugation and washed twice with FlowBuffer-1. Secondary antibody Alexa 488 was added at a concentration of 1:2000 in FlowBuffer-2 and incubated in the dark at room temperature for 30 min. Then, the excess of secondary antibody was removed by centrifugation and the addition of FlowBuffer-1 two times. Cells were dissociated in a 1.5 mL tube with FlowBuffer-1 and analyzed by flow cytometry.

### 2.10. Statistical Analysis

A *t*-test was performed to verify the measures of LCST with the three different methods. For cytotoxicity analysis of NIH-3T3 on terpolymer, a nonparametric test was performed. Additionally, a Tukey Multiple comparison test was employed to evaluate the sizes of SKOV-3 spheroids inside terpolymer scaffolds and cell viability and expansion of SKOV-3 and Jurkat T cells. The calculated ^1^H-NMR molar composition and the average number of molecular weight results were statistically compared by Grubbs’ Test for Outliers detection. These studies were executed in Minitab 19 and Prisma 9 programs using a 5% significance level.

## 3. Results and Discussion

### 3.1. Spectroscopic Analysis

**Figure 2:**
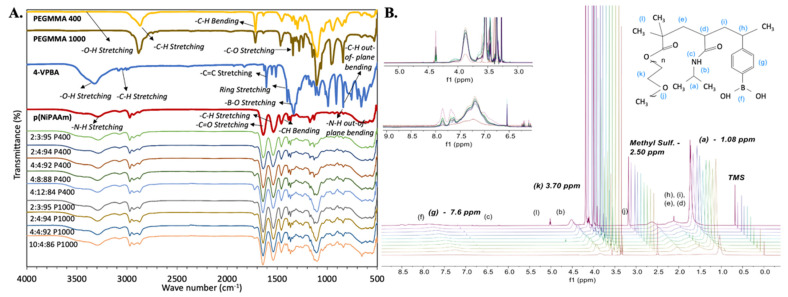
Terpolymer spectrometric characterization. (**A**) FT-IR spectra of synthesized terpolymers, including pNiPAAm as a baseline. (**B**) ^1^H-NMR spectra with identification of all corresponding protons in the polymer, including pNiPAAm as a baseline. After pNiPAAm, synthesized terpolymers spectrum are shown from bottom to top as follows: 2:3:95, 2:4:94, 4:4:92, 4:8:88, and 4:12:84 P400 terpolymers, then 2:3:95, 2:4:94, 4:4:92, and 10:4:86 P1000 terpolymers.

FT-IR and ^1^H-NMR techniques were employed to confirm the incorporation of all monomers during polymerization. pNiPAAm samples were used as a baseline to evaluate FT-IR and ^1^H-NMR spectra. The presence of all expected chemical moieties and protons was identified in the structure of the polymer, as observed in Figure 2. 

#### 3.1.1. FT-IR

As expected, FT-IR results indicated that the pNiPAAm spectrum dominates in all terpolymer combinations. However, the presence of functional groups from VPBA and PEGMMA monomers demonstrated the success of polymerization. 

In this spectroscopic analysis, acrylates and ether stretching vibrations of the PEGMMA monomer were also found, including -C-H stretching at 2850 cm^−1^, -C=O stretching at 1700 cm^−1^, and -C-O stretching at 1100 cm^−1^. Strong -B-O stretching at 1320 cm^−1^ in PBA linkage and the single strong deformation band for the benzene ring, -C-H out-of-plane bending at 850 cm^−1^ of the VPBA monomer were also identified. Table 1 summarizes characteristic FT-IR band wavelengths reported in the literature [56,63,64,65,66,67,68,69,70] for the functional groups and the corresponding measured bands of the synthesized terpolymers. Differences in the intensity of the FT-IR peaks also illustrate differences between terpolymers according to their composition (See Figure 2).

#### 3.1.2. ^1^H-NMR

^1^H-NMR spectra showed characteristic resonance signals of all protons present in the terpolymers structure. Like FT-IR results, ^1^H-NMR results indicated that the spectrum of pNiPAAm dominates in all terpolymer combinations. However, the protons of VPBA and PEGMMA monomers were also identified in the terpolymer samples (see Figure 2). Protons located in the terminals of each monomer were especially identified for further analysis, including 2 (CH_3_) terminals of NiPAAm at ~1.0 ppm, 2 (CH_2_) of repetitive units of PEG at ~3.5 ppm, and 4 (CH) of the ring terminal of VPBA at ~7.6 ppm [56,57].

##### Polymer Molar Composition

The molar composition of the terpolymers (mol%) was calculated using the area under the terminal peaks of the ^1^H-NMR spectra normalized with the total number of protons present on each peak. The calculated average ^1^H-NMR mol% and standard error of the mean (SEM) results of six samples were compared between replicates and the actual mol% used in the polymerization reaction. The summary of mol% results is illustrated in Table 2, where the calculated molar composition was compared with the actual mol% of terpolymer combinations. Results were statistically compared using Grubbs’ Test to detect outliers, indicating that the calculated mol% does not have a significant statistical difference between reactor replicates in all terpolymer combinations. In addition, when the amount of VPBA and PEGMMA was small (e.g., 2:3:95 P400 and 2:4:94 P400 terpolymers), the feed composition was similar to the molar composition of the resulting polymer. By contrast, terpolymer combinations with a higher amount of VPBA and PEGMMA, such as 10:4:86 P1000 terpolymer, yielded different mol% than the actual molar composition used. Apparently, incorporating the PEGMMA monomer in the terpolymer composition was more favorable than the 4-VPBA monomer. This behavior was observed in terpolymer combinations fed with more than 2 mol% of 4-VPBA monomer, for which the expected values were not reached. This interaction could be explained by the behavior of the NiPAAm/VPBA copolymer previously reported by Rzaev et al. In that study, the NiPAAm/VPBA copolymer behavior was compared to the NiPAAm/styrene copolymer model. They concluded that the NiPAAm/VPBA copolymer has a tendency to possess an alternating structure with a predominant arrangement of NiPAAm macroradicals and VPBA monomer additions to the copolymer chain [63]. For terpolymers with an actual mol% of 4% VPBA, the resulting composition after polymerization was 2.66 ± 0.15, 2.16 ± 0.07, and 2.84 ± 0.13 mol% for 4:4:92 P400, 4:8:88 P400, and 4:4:92 P1000 terpolymer combinations, respectively. Even when the maximum amount of 4-VPBA in the fed was 10.0 mol% (10:4:86 P1000 terpolymer), the 4-VPBA mol% obtained in the terpolymer was only 4.18 ± 0.09 mol%. These results of monomers molar composition in the terpolymer are important since the amount of 4-VPBA is responsible for the potential attachment of biological molecules to the terpolymer scaffolds. 

##### Molecular Weight (*M_n_*)

The molecular weight of the resulting polymer was determined using ^1^H-NMR. Data obtained from ^1^H-NMR spectra allowed the calculation of *M_n_* based on end-group analysis, which uses the integral area of the aforementioned end-species moieties. The resonance signal of those end-species is proportional to its concentration. It can also be compared with the chain repeating units (degree of polymerization, DP) in the terpolymer structure. Analyzing the chemical structure of the monomers conforming to the terpolymer molecule, the only monomer whose repetitive unit is known is the PEGMMA monomer. Therefore, PEGMMA repetitive units (DPs) in the terpolymer were calculated based on one PEGMMA molecule, which has an average of 9 or 23 repetitive units for the 400 and 1000 molecular weights, respectively. This DP of PEGMMA allowed the proportional calculation of 4-VPBA and NiPAAm DPs using Equation 2 described in the methodology section. Table 2 summarizes the estimated *M_n_* calculated by ^1^H-NMR, where all moieties in the terpolymer structure were considered and multiplied by their respective DP. In the case of the PEGMMA400 terpolymers, it was observed that *M_n_* values were inversely proportional to the amount of PEGMMA monomer; the lower the PEGMMA content, the higher the *M_n_*. Nevertheless, the *M_n_* values of 2:4:94 and 4:4:92 terpolymer combinations of both PEGMMA molecular weights do not have statistical differences. Both behaviors were also observed in 2:3:95, 2:4:94, and 4:4:92 PEGMMA1000 terpolymer combinations. The relationship between *M_n_* and NiPAAm/PEG content was in agreement with the results of a previous NiPAAm-PEG block copolymerization study developed by Kwon et al. [64]. They found that the NiPAAm molar composition of copolymers has a linear relationship with *M_n_*, which increased as the amount of NiPAAm was increased in the polymer composition. Copolymers with NiPAM:PEG molar ratios of 100, 500, 1000, and 2000 yielded polymers with an *M_n_* of 0.9, 2.2, 5.6, and 9.3 × 104 g/mol, respectively [64]. This behavior could be attributed to higher amounts of polar hydroxyl end-groups, and reactive end-groups as the amount of PEGMMA monomer was increased in the terpolymer composition [65,66]. The higher number of reactive end-groups generates more molecules per volume of sample, resulting in a lower *M_n_*.

### 3.2. Thermal Sensitivity

**Figure 3:**
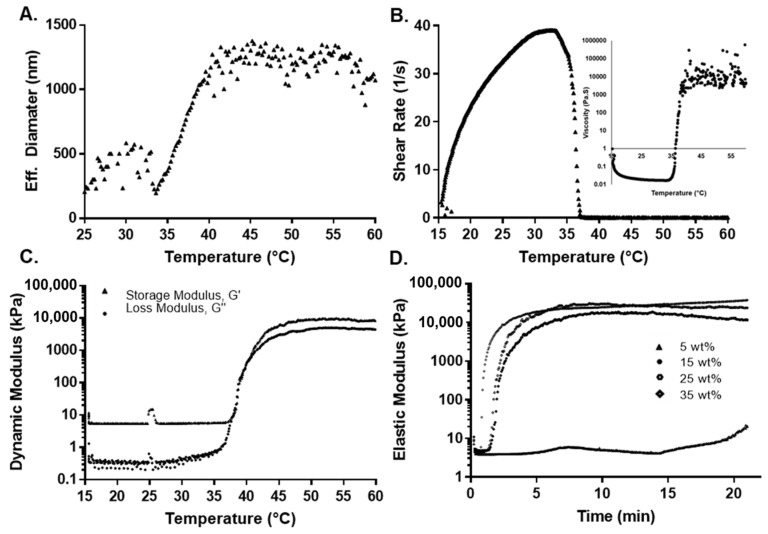
Thermal and mechanical characterization for 2:4:94 P400 terpolymer. (**A**) Effect of temperature on effective diameter measured by DLS. (**B**) Shear rate and viscosity as a function of temperature. (**C**) Rheological measurement of the dynamic modulus as a function of temperature. (**D**) Oscillatory rheological determination of the elastic modulus as a function of concentration at 37 °C.

The determination of pNiPAAm LCST in an aqueous solution is commonly determined using ultraviolet-visible (UV-Vis) spectroscopy, dynamic light scattering (DLS), or rheological measurements [71]. Due to the terpolymer translucency, the UV-Vis technique was not used in this study. DLS provided information about the translational diffusion coefficient that is converted to hydrodynamic diameter with the Stoke–Einstein relation. This information, then, provides the means to relate the size of the agglomerated polymers with their thermal properties, as the chains are mostly extended when the terpolymer is below the LCST and vice versa [72,73]. The behavior between the hydrodynamic diameter of the 2:4:94 P400 terpolymer versus temperature is shown in Figure 3A. Supplementary Information, Figure S1 presents the result for all terpolymers’ combinations. As observed from Figure S1, the hydrodynamic diameter of pNiPAAm increased suddenly due to the LCST (31 to 33 °C). In the case of terpolymers, the LCST curve was much more elongated due to the influence of PEGMMA and its hydrophilicity, which generated an increase in the transition phase of the terpolymer [74,75]. For the terpolymer with PEGMMA400, the hydrophobic behavior of the VPBA can be clearly appreciated. VPBA decreased the LCST, which was opposite to the PEGMMA effect [45,76,77]. Table 3 provides a summary of the results obtained from the DLS analysis.

For 2:3:95 P400, 2:4:94 P400, and 4:8:88 P400, results indicated that the LCST increased with the amount of PEGMMA. However, for 4:4:92 P400, it decreased since it has a higher percentage of VPBA in the synthesis compared to other compositions. Conversely, for 4:12:84 P400, which contains a higher amount of PEGMMA400 and a lower proportion of NiPAAm monomer, the LCST was significantly higher. Finally, the 2:4:94 P400 terpolymer had the LCST closest to body temperature.

For the case of terpolymer combinations with PEGMMA1000, the LCST was lower with respect to the synthesis with PEGMMA400 under similar molar compositions. It is important to note that, although both combinations have the same molar proportion of each monomer, the NiPAAm/PEG unit ratio for PEGMMA400 is different. The PEGMMA 1000 has over 224% more PEG units when compared to the NiPAAm/PEG400 unit. This value can explain the higher LCST for PEGMMA1000 terpolymers due to the PEGMMA effect in the synthesis. The 10:4:86 P1000 has the lowest LCST for the highest quantity of VPBA. In addition, the large chains of PEGMMA1000 may affect the effectiveness of the other monomers.

The thermal stability of the 2:3:94 P400, 2:4:94 P400, and 10:4:96 P1000 terpolymers was studied by TGA and compared with synthesized pNiPAAm (Supplementary Information, Figure S2). For all cases, TGA analysis presented two stages of degradation for pNiPAAm and terpolymers with an increase in temperature. The first stage was a 12% weight loss as the solution was heated from 25 to 150 °C. This initial loss can be attributed to the evaporation of remanent water in the polymeric matrix. The second major weight loss stage was seen from 330 to 420 °C due to the backbone degradation of terpolymer. These results revealed that the terpolymer was thermally stable at body temperature.

### 3.3. Rheological Measurement

In addition, two rheological tests, (1) constant-stress viscosity and (2) dynamic oscillatory shear tests as a function of temperature, were used to investigate the thermal transition of the copolymers. It has been reported that polymer solutions are very sensitive to shear rate around their phase transition [78].

A dynamic oscillatory shear test was used to determine the transition temperature by identifying the temperature at which the storage and loss modulus are equal (i.e., solid- and liquid-like contributions are equal). Initially, as the temperature was increased during the constant-stress viscosity test, the viscosity decreased, which was attributed to an increase in thermal motion. To maintain constant stress, the rheometer compensated by reducing the shear rate to a value close to zero, while predicting a substantial viscosity. Figure 3B shows this behavior for the 2:4:94 P400 terpolymer. This inflection in behavior was attributed to the structural changes around the LCST. The dynamic oscillatory shear test (shown in Figure 3C for 2:4:94 P400 and Figures S3–S7 of the Supplementary Information for the rest of the synthesis) indicated that the storage or elastic modulus (G′) was higher than the loss or viscous modulus (G″) even at lower temperatures. This behavior can result from polymer interactions, since the concentration of the polymer solution can be considered between moderate to the concentrated range [79]. While a crossover from liquid-like behavior (G″ > G′) to solid-like (G′ > G″) behavior could not be clearly identified, sharp increases in both moduli were observed. The elastic component (G′) remained above the viscous component (G″) and reached values of above 1 to 10 kPa at higher temperatures, at least three to four orders of magnitude higher than values below room temperature. This behavior also implies the formation of a network spanning the whole sample. The LCST with the dynamic method was calculated by the first crossover of (G′) and (G′′). The LCSTs determined by both methods are summarized in Table 3.

These results also support the observation from the DLS analysis, where the LCST changes due to the presence of VPBA and PEGMMA, explained by hydrophobic-hydrophilic interactions. Near the LCST point, the viscosity and moduli rapidly increased with temperature. This is due to intermolecular aggregation caused by the LCST effect, which depends on the polymer concentration. Beyond LCST, there is a higher phase separation that is unstable as temperature increases [80].

The effect of monomer composition on the mechanical properties was evaluated by measuring the dynamic elastic modulus (G′) over a 20 min span at a constant temperature of either 37 or 45 °C at a constant strain of 5% and frequency of 2 Hz. Fresh samples were used to eliminate the effect of any previous thermal history. The elastic modulus increased over the first 4 min, until it reached a plateau value. This value remained unaffected over the experimental window, as shown in Figure 3D. The plateau values of the elastic moduli for all copolymers at 37 and 45 °C are summarized in Table 3. For all cases, the elastic modulus was higher at 45 than 37 °C, because the polymers exceeded their LCST. A gel network has completely formed at this point, making the sample mechanically stronger. Terpolymers with a low amount of NiPAAm presented a low elastic modulus (e.g., 4:8:88 P400 and 4:12:84 P400 with an elastic modulus of 2.9 ± 0.5 and 0.5 ± 0.2 kPa, respectively), due to the reduction of strength contribution of NiPAAm monomer. The inclusion of VPBA and PEGMMA affected the moduli compared to pure pNiPAAm due to the decreased thermo-responsive component. In the case of terpolymers with PEGMMA400 monomer, the mechanical properties were higher with respect to the same combination with PEGMMA1000 (e.g., the combination 2:3:95 showed a modulus of 19.1 ± 3.3 kPa with PEGMMA 400 and 0.3 ± 0.1 kPa with PEGMMA1000). This behavior indicated that the mechanical properties decreased significantly for larger chains of PEG [81].

These polymer combinations present a range of moduli within the storage moduli of several types of tissues, especially the ones used in cell therapies [82,83]. For example, the reported storage modulus for human cardiac tissue is between 10 and 15 kPa [84]. In the case of the lymphoid tissues, stiffnesses between 0.1 and 2 kPa [6,85] can be found. Finally, for mesenchymal stem cells (MSCs), osteogenesis can occur at stiffnesses of 20 kPa [86]. Therefore, the polymer combinations described herein exhibit mechanical properties similar to several known tissues and could potentially be used for their culture.

### 3.4. Terpolymer Testing

#### 3.4.1. Cell Viability

Once characterized, washed, and sterilized, terpolymer samples were ready for cell culture testing. After the washing process, samples were dissolved in cell culture media. No changes in pH were observed, indicating that potential effects on the cells were not the result of pH changes. Terpolymer samples were sterilized by filtration using PES membranes showing no significant weight loss before and after the filtration (Section 3 of Supplementary Information). Terpolymer combinations 2:3:95 P400, 2:4:94 P400, 4:8:88 P400, 4:12:84 P400, and 10:4:86 P1000 were selected as the most promising combinations for cell culture testing in multiple cell lines, due to the transparency, gel behavior, stiffness of the system, and LCST behavior.

A mouse fibroblast (NIH-3T3) cell line was used as a standard to test the cytotoxicity of 2:3:95 P400, 2:4:94 P400, and 10:4:86 P1000 terpolymers [87]. The 2:4:94 P400 was selected as its LCST is close to body temperature. A second combination, 10:4:86 P1000, was selected for its transparency. These were used to test the viability of the terpolymer combinations using the NIH-3T3 cells.

**Figure 4:**
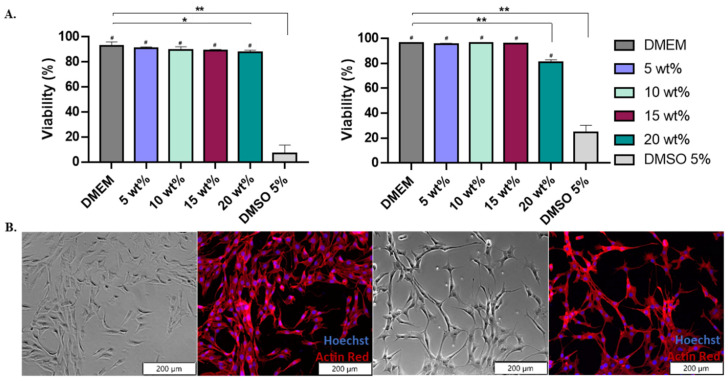
Terpolymer cell viability using NIH-3T3 cell line. (**A**) The general trend of 2:4:94 P400 (**left**) and 10:4:86 P1000 (**right**) cell viability (%) test of NIH-3T3 cell line for an exposure time of 24 h with concentrations of 5, 10, 15, and 20 wt.% of terpolymer in cell culture media for n = 12. *p*-values: 0.0383 (* versus DMEM), <0.0001 (** versus DMEM) and <0.0001 (# versus DMSO). (**B**) Brightfield and fluorescent images of NIH-3T3 cell line cultured on tissue culture plate (**left** pictures) and 2:4:94 P400 terpolymer at 15 wt.% (**right** pictures). Pictures show cells live at 24 h of incubation. The fluorescent image shows the stained cytoskeleton with Actin Red (red color) and the cell nucleus with Hoechst (blue color).

Cells were exposed for 24 h to various terpolymer concentrations in culture media (5, 10, 15, and 20 wt.%). The viability of NIH-3T3 cells after 24 h of incubation with 2:4:94 P400 and 10:4:86 P1000 terpolymer demonstrated that cells survived and proliferated on the hydrogel. There was no significant cytotoxicity within the range of tested concentrations (5, 10, and 15 wt.%) with respect to the control DMEM. Significant toxicity was found when using DMSO at 5%, as expected. In the case of 20 wt.%, it presented a significant reduction with respect to the DMEM, as reported by *t*-test analysis. However, this condition did not represent a high level of cytotoxicity. Figure 4 also illustrates that cell observation by microscopy was possible, and no polymer interference was observed.

**Figure 5:**
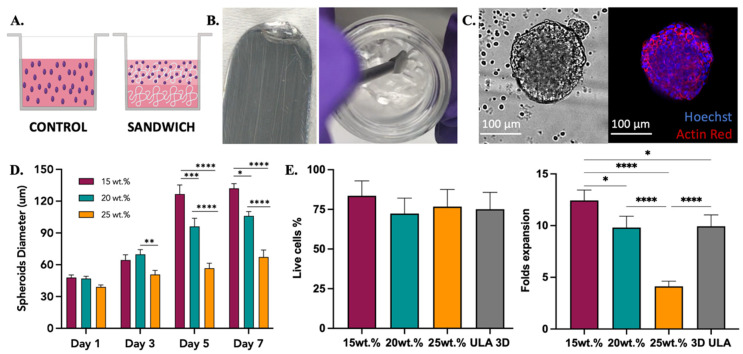
SKOV-3 cell culture testing with 2:4:94 P400 terpolymer combination. (**A**) Conditions employed to test terpolymer scaffolds with SKOV-3 cell culture. Control: Cells in the same working volume as other conditions. Sandwich: Mixing of cells with terpolymer seeded on top of a terpolymer coating. SKOV-3 cells were tested with 2:4:94 P400 terpolymer using the sandwich condition. (**B**) Transparency of 2:4:94 P400 terpolymer combination. (**C**) Brightfield and merge fluorescence pictures of SKOV-3 cells after 10 days of culture. 2D Control (**right** side) and spheroid growth (**left** side) inside 15 wt.% terpolymer scaffolds. The blue color represents the nucleus of cells, while the red color represents the cytoskeleton, which was stained with Hoechst and Actin Red staining solutions, respectively. Scale bar: 100 µm. (**D**) The general trend of encapsulated spheroids size over the days 1, 3, 5, and 7 of cultivation inside 15, 20, and 25 wt.% terpolymer scaffolds. (**E**) Cell viability and live cell fold expansion of harvesting after 7 days of encapsulation. The graphs show means and STDV values of n = 6 spheroids, except for cell viability which expresses error propagation. *p*-values: blank (not significant), 0.0078 (*), 0.0057 (**), 0.0003 (***), <0.0001 (****).

**Figure 6:**
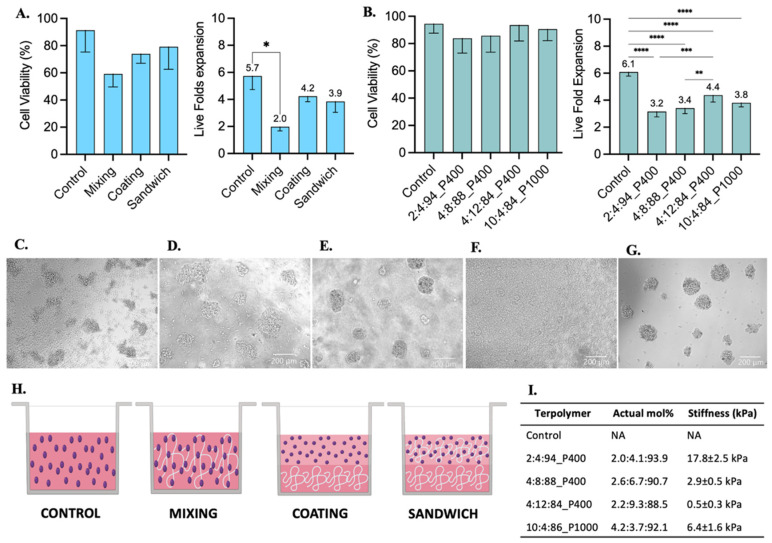
Jurkat T cell culture testing with 2:4:94 P400, 4:8:88 P400, 4:12:84 P400, and 10:4:86 P1000 terpolymer combinations after 48 h of incubation. (**A**) Cell viability and live fold expansion using mixing, coating, and sandwich conditions of terpolymer scaffolds to test 2:4:94 P400 terpolymer. (**B**) Cell viability and live fold expansion using the sandwich condition with different terpolymer combinations. Brightfield pictures of (**C**) suspension cell culture as control, encapsulated cells within (**D**) 2:4:94 P400, (**E**) 4:8:88 P400, (**F**) 4:12:84 P400, and (**G**) 10:4:86 P1000. Scale bar: 200 µm. (**H**) Graphical representation of conditions employed to test terpolymer scaffolds with Jurkat T cell culture. Control: Cells in the same working volume as other conditions. Mixing: mix of 1:1 proportion of terpolymer solution with cells in suspension. Coating: Cells seeded on top of a previously made polymer-coated well. Sandwich: Mixing of cells with terpolymer seeded on top of a terpolymer coating. (**I**) Summary of principal characteristics of terpolymer tested with Jurkat T cells. The graphs show means and STDV values of n = 6 wells, except for cell viability which expresses error propagation. *p*-values: blank (not significant), 0.0078 (*), 0.0057 (**), 0.0003 (***), <0.0001 (****).

The viability in other cell lines was also tested, including the SKOV-3 and Jurkat T cell lines. Results indicated that there were no statistical differences between the control and the tested terpolymers, as depicted in Figure 5E and Figure 6A,B. These analyses confirmed that several synthesized terpolymers are suitable for mammalian cell culture.

#### 3.4.2. SKOV-3 Cell Line

The ovarian cancer SKOV-3 cell line was used as a model for cell encapsulation, spheroid formation, and 3D culture. SKOV-3 cells were grown in the presence of 15 and 20 wt.% scaffolds of 2:3:95 P400, 2:4:94 P400, and 10:4:86 P1000 terpolymers. Two methods to encapsulate SKOV-3 cells were explored, mixing and sandwich conditions. In the mixing condition, cells were mixed with the polymer and seeded, while in the sandwich condition, a layer of the polymer was created first, and then the mixed cells were seeded on top, as described in Figure 5A. The transparency of terpolymer scaffolds including cells was reached after a few seconds of taking them out of the incubator, and it was corroborated when cells were monitored. The gel phase and transparent matrix of 2:4:94 P400 terpolymer could also be observed in Figure 5B. Similar to NIH-3T3, SKOV-3 cells were observed by microscopy through the transparent terpolymer scaffolds without difficulty. It was observed that when the mixing condition was used, cells appeared to have fallen to the bottom of the well before gelation was obtained, resulting in a 2D growth on the well surface. The 2D cell growth of SKOV-3 cells is shown in Supplementary information Figure S8. The sandwich condition prevented such problems, thus achieving the successful formation and encapsulation of SKOV-3 spheroids with different concentrations (15, 20, and 25 wt.%) of the 2:4:94 P400 terpolymer scaffold.

Multiple spheroid formation was achieved when the sandwich condition was employed. Figure 5C illustrates an example of the spheroids obtained. The general trend of SKOV-3 spheroids sizes over time inside 2:4:94 P400 scaffolds is illustrated in Figure 5D. Spheroid diameter size analysis was performed with ImageJ software with the scale bar tool [88]. These results indicated that polymer concentration appears to affect the resulting spheroid size (Figure 5D). The cell viability was the same in all tested conditions, including the 3D control spheroid cultured in a round bottom ultra-low attachment plate. Moreover, the cell viability and expansion illustrated in Figure 5E showed that, similar to spheroid sizes, cell expansion was affected by the concentration of the terpolymer. As the concentration of the polymer increased, the size of the resulting spheroid and cell growth was smaller when compared to lower concentrations at the same time point. This behavior was expected as the spheroid morphology, and cell adhesion can be affected by the mechanical properties of the scaffolds [89,90]. In addition, Figure 3D illustrates how the terpolymer concentration influences the stiffness of the scaffold. Previous studies of SKOV-3 cell behavior in bioengineered 3D platforms with stiffness ranging from 0.24 to 1.2 kPa [91] and 1.4 to 62 kPa [92] also confirmed that material stiffness influences size, cell migration, and spheroid formation [91,92]. Moreover, the multiple spheroids formation observed within the polymer matrix was also reported by Loessner et al. They used synthetic PEG-based hydrogels with attached RGD peptides that possessed a stiffness between 0.24 to 1.2 kPa [91]. Unlike these synthetic hydrogels, the terpolymer matrix was not functionalized with peptides to simulate tumor ECM interactions. The scaffolds of 2:4:94 P400 terpolymer used for SKOV-3 cell experiments, most likely permitted cell migration and subsequent agglomeration through the terpolymer matrix. The successful results of SKOV-3 cell growth, spheroid formation, and encapsulation inside terpolymer scaffolds, confirmed the potential use of the terpolymer scaffolds as 3D matrices for cell culture applications.

#### 3.4.3. Jurkat T Cell Line

Jurkat T cells were selected as a model for the culture of suspension cells within synthesized terpolymers. These cells were cultured with the terpolymer scaffolds using the coating, sandwich, and mixing conditions. Jurkat T cells were able to successfully grow in all tested conditions. A significant difference was only found when cells were cultured using the mixing condition when compared with suspension control, see Figure 6A. The Jurkat T cell viability between coating, sandwich, and mixing conditions had no significant differences with respect to suspension control.

Furthermore, similar to SKOV-3 cells, Jurkat T cells were successfully encapsulated in 3D within 7.5 wt.% scaffolds of 2:4:94 P400, 4:8:88 P400, 4:12:84 P400, and 10:4:86 P1000 terpolymers. Encapsulated cells in all terpolymer combinations were able to grow and behave like the control suspension, forming cell clusters after encapsulation (Figure 6D–G). The transparency of terpolymer matrices was also corroborated with brightfield pictures depicted in Figure 6, except for 4:12:84 P400 terpolymer (Figure 6F), where cells can hardly be seen. Nevertheless, the 4:12:84 P400 scaffold was the one that got the higher Jurkat T cell expansion. The lymphoid tissues, where T cells are naturally expanded in the human body, possess a stiffness value lower than 2 kPa [6,85], which agreed with the preference of Jurkat T cells to grow better in 4:12:84 P400 with a stiffness value of 0.5 ± 0.3 kPa.

#### 3.4.4. iPSC Cell Line

**Figure 7:**
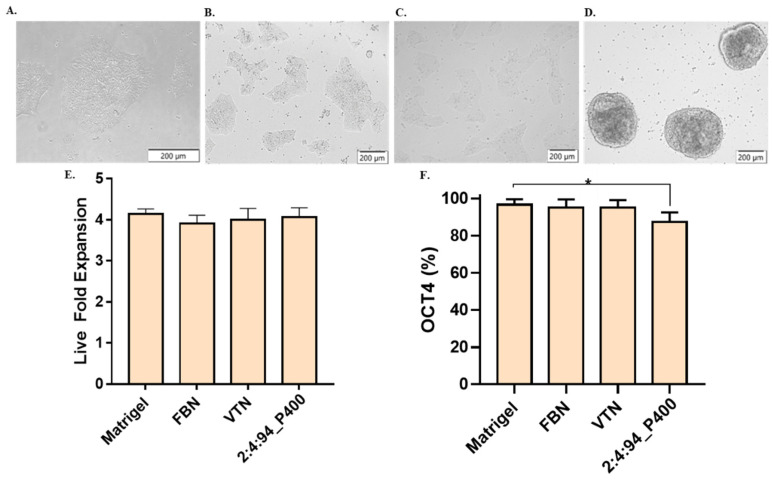
iPSCs cell culture testing. Brightfield pictures of WTC-11 cultured over a coating of (**A**) Matrigel, (**B**) Fibronectin, (**C**) Vitronectin xF, and (**D**) 2:4:94 P400 terpolymer. (**E**) Live fold expansion time for cells cultured on a layer of terpolymer. (**F**) iPSCs pluripotency measured by Oct-4 in flow cytometer for same conditions. Controls: Cells seeded at the same conditions on Matrigel, Fibronectin (FBN), and Vitronectin xF (VTN). *p*-values: 0.0488 (*).

Induced pluripotent stem cells from passage 50 were tested to verify the potential application of the terpolymer in the growth and pluripotency of these cells. Similar to the NIH-3T3, SKOV-3, and Jurkat T cells, the 2:4:94_P400 combination was selected for its transparency and gelation close to body temperature. A number of 100,000 cells/cm^2^ were seeded, and the amount of terpolymer was fixed at 15 wt.% to cover the entire surface of the plate. The dish was previously coated with terpolymer. Matrigel, Fibronectin, and Vitronectin xF were selected as control. Figure 7A,D displays the culture of WTC-11 in Matrigel and terpolymer at 15 wt% on day 3. It was evident that cell morphology changed in the presence of the terpolymer, which is quite similar to iPSC spheroids cultured in 3D [93,94]. In addition, cell monitoring was possible by microscopy due to the transparency of the terpolymer. In terms of proliferation, the fold expansion was calculated and presented in Figure 7E. The WTC-11 cells showed a fold change similar to the fold expansion of the Matrigel, Fibronectin, and Vitronectin xF controls.

Flow cytometry was used to assess the expression of the Oct-4 transcription factor, which was used to identify whether the cell was pluripotent. This factor is essential in investigating stem cell differentiation in regenerative medicine. Results indicated that pluripotency remained at high levels. This successful result of high levels of iPSCs pluripotency confirmed the possible use of the terpolymer for later iPSCs differentiation [95,96]. To further improve the culture of iPCSs on the terpolymer matrix, the addition of biological motifs such as RGD, RGDS, or Fibronectin peptides was investigated based on iPSC binding to Matrigel to facilitate the activation of several integrin pathways and promote cell adhesion and expansion.

## 4. Conclusions

In this study, free radical polymerization successfully synthesized a synthetic, transparent, and thermo-responsive synthetic polymer for the culture and encapsulation of different cell lines. Several polymer characterization techniques were employed to determine the physico-chemical properties of the synthesized materials. First, the FT-IR and ^1^H-NMR spectra were analyzed to confirm the successful synthesis by identifying all the components of the used monomers. Within FT-IR and ^1^H-NMR spectra of synthesized terpolymers, it was observed that PEG and 4-VPBA monomers were successfully incorporated. In addition, the monomer molar composition and terpolymers molecular weight (*M_n_*) were determined by ^1^H-NMR analysis.

Additionally, DLS and rheological analysis were performed to understand the thermal and mechanical properties of the terpolymers combinations. DLS, rotatory, and oscillatory measurements confirmed the terpolymers LCST. The stiffness was measured by oscillatory analysis in the rheometer. These results showed that LCST and stiffness depended on the ratio of hydrophobic/hydrophilic monomers incorporated in the chain. Hydrogels with LCST close to body temperature and stiffness similar to the ECM of different tissues were found. These combinations were tested in 2D and 3D cultures with NIH-3T3, SKOV-3, iPSC, and Jurkat T cell lines. After culture, cells were successfully harvested from the terpolymer scaffolds for further analysis. SKOV-3 cells were successfully cultured in 3D with 2:4:94 P400 terpolymer using the sandwich condition, achieving more cell growth than 3D control, which was formed in a round-bottom ultra-low attachment plate. In addition, the Jurkat T cell culture results using 2:4:94 P400, 4:8:88 P400, 4:12:84 P400, and 10:4:86 P1000 terpolymers demonstrated that they possess potential features for the proper T-cell encapsulation and expansion. NIH-3T3 cell viability showed that cells survived and proliferated in the 2:4:94 P400 and 10:4:86 P100 combinations. Cell culture results of these cell lines and cell cytotoxicity with NIH-3T3 validated that terpolymers exhibit transparency, which allows cell monitoring through microscopy. Tested terpolymers also appeared to promote cell aggregation, attachment, and encapsulation within the tested platforms. iPSC cell culture and pluripotency analysis showed the potential application of the terpolymer platform for the maintenance of stem cells.

Furthermore, these results demonstrated that the molecular design of monomers used in the synthetic terpolymer combinations was effective from the synthesis to their application in cell culture. In addition, they were synthesized using a simple fabrication technique. Cell culture experiments were performed multiple times with different terpolymer batches, confirming that the polymerization synthesis process was reproducible. This tunable hydrogel is attractive for cell culture applications due to its well-defined properties.

## 5. Patents

This work was registered as an invention disclosure under the University of Puerto Rico with ID 21-002-DISC-UPR and international patent application number PCT/US2021/061256, identified as Synthetic Matrixes for Cell Culture and Manufacture.

## Figures and Tables

**Table 1:** Summary of characteristic functional groups of monomers identified in the FT-IR spectra of p(NiPAAm-co-4-VPBA-co-PEGMMA) terpolymers.

Functional Groups	Monomer	Wavelength Reference (cm^−1^)	Terpolymers (cm^−1^)
-N-H stretching [56,70]	NiPAAm	~3400–3300	~3350
-C-H stretching [69]	PEGMMA	~2700–2950	~2850
-C=O stretching [65,68]	PEGMMA	~1750	~1700
-C=O stretching [56,70]	NiPAAm	~1650	~1600
-C-H stretching [56]	NiPAAm	~1605	~1500
-C-H bending [56]	NiPAAm	~1475	~1450
-B-O stretching [64]	4-VPBA	~1550–1300	~1320
-C-O stretching [66]	PEGMMA	~1100	~1100
-C-H out-of-plane bending [64,70]	4-VPBA	~870–650	~850

**Table 2:** Average results and standard error of the means (SEM) of number average molecular weight (*M_n_*) obtained from ^1^H-NMR spectra. The size of the samples was n = 6 unless otherwise stated.

Syntheses	*M_n_* (gr/mol)	Mol %	^1^H-NMR Molar Composition%		
			4-VPBA	PEGMMA	NiPAAm
2:3:95 P400 *	3353 ± 253	Actual	2.0	3.0	95.0
		^1^H-NMR	2.3 ± 0.1	3.0 ± 0.1	94.7 ± 0.1
2:4:94 P400 *	2596 ± 185	Actual	2.0	4.0	94.0
		^1^H-NMR	2.0 ± 0.1	4.1 ± 0.3	93.9 ± 0.2
4:4:92 P400 *	2836 ± 132	Actual	3.9	3.9	92.2
		^1^H-NMR	2.7 ± 0.2	3.6 ± 0.1	93.7 ± 0.1
4:8:88 P400	1661 ± 31	Actual	3.8	7.5	88.7
		^1^H-NMR	2.6 ± 0.1	6.7 ± 0.1	90.7 ± 0.1
4:12:84 P400	1234 ± 6	Actual	3.6	10.9	85.5
		^1^H-NMR	2.2 ± 0.1	9.3 ± 0.1	88.5 ± 0.1
2:3:95 P1000	4061 ± 186	Actual	2.0	3.0	95.0
		^1^H-NMR	1.7 ± 0.1	3.0 ± 0.1	95.3 ± 0.1
2:4:94 P1000	3125 ± 150	Actual	2.0	4.0	94.0
		^1^H-NMR	1.8 ± 0.1	3.6 ± 0.1	94.6 ± 0.1
4:4:92 P1000	3316 ± 102	Actual	3.9	3.9	92.2
		^1^H-NMR	2.3 ± 0.1	3.7 ± 0.1	93.9 ± 0.2
10:4:86 P1000	2690 ± 93	Actual	9.3	3.7	87.0
		^1^H-NMR	4.2 ± 0.1	3.7 ± 0.1	92.1 ± 0.1

* Five reactors were used during the polymerization reaction.

**Table 3:** Summary of thermal and mechanical properties of the different p(VPBA-co-PEGMMA-co-NiPAAm) terpolymers and pure pNiPAAm. LCST was determined by DLS, constant-stress temperature ramp (CSTR) viscosity measurements, and dynamic oscillatory shear measurements. Stiffness was measured by oscillatory experiment at 37 and 45 °C. Average and variation calculated with n = 6, except for synthesis 2:3:95 P400, 2:4:94 P400, 4:4:92 P400, and 4:8:88 P400, where the sample number was n = 5. Average and variation for stiffness calculated with n = 3. The *t*-test result does not present a significant variation between the three different methods to calculate LCST.

Polymer	LCST (DLS, °C)	LCST (CSTR, °C)	LCST (Dynamic, °C)	Elastic Modulus, G′ (kPa) @37 °C *	Elastic Modulus, G′ (kPa) @45 °C *
pNiPAAm	31.5 ± 0.3	31.2 ± 0.2	31.6 ± 0.4	16.3 ± 2.4	-
2:3:95 P400	34.9 ± 0.3	34.9 ± 0.9	34.5 ± 0.9	19.1 ± 3.3	20.9 ± 2.6
2:4:94 P400	36.5 ± 0.7	37.2 ± 1.7	37.3 ± 0.4	17.8 ± 2.5	21.6 ± 2.5
4:4:92 P400	33.6 ± 0.7	34.8 ± 1.5	33.8 ± 0.7	13.6 ± 1.3	14.4 ± 1.5
4:8:88 P400	42.3 ± 0.7	41.4 ± 1.3	40.6 ± 0.8	2.9 ± 0.5	2.2 ± 0.2
4:12:84 P400	44.4 ± 0.8	44.9 ± 2.2	43.2 ± 0.6	0.5 ± 0.2	2.2 ± 0.4
2:3:95 P1000	44.2 ± 0.6	44.7 ± 2.1	45.2 ± 0.8	0.3 ± 0.1	0.7 ± 0.1
2:4:94 P1000	43.2 ± 0.6	42.0 ± 1.2	43.8 ± 0.6	1.5 ± 0.4	3.3 ± 0.5
4:4:92 P1000	44.1 ± 0.3	44.1 ± 1.8	44.1 ± 0.7	0.5 ± 0.1	2.6 ± 0.2
10:4:86 P1000	42.8 ± 0.7	43.5 ± 1.0	43.1 ± 0.5	6.4 ± 1.6	7.5 ± 0.8

* Constant strain 5% and frequency 5 Hz.

## Supplementary Materials

The supporting information can be downloaded at: https://www.mdpi.com/article/10.3390/polym14204379/s1. Figure S1: Effect of temperature on effective diameter of pNiPAAm as baseline and terpolymer, Figure S2: Thermal gravimetric analysis for pNiPAAm pure, 2:3:95_P400, 2:4:94_P400 and 10:4:86_P1000 terpolymer combinations, Figures S3–S7: Dynamic modulus in function of temperature for pNiPAAm and therpolymer at 15 wt.%, Figure S8: Mixing condition used to encapsulate SKOV-3 ovarian cancer cell line within terpolymer scaffolds, Figure S9. Pluripotency plots for Matrigel, Fibronectin (FBN), Vitronectin (VTN), and 2:4:94 P400 terpolymer combination, Figures S10–S12: T-Test analysis in Minitab software of wet and dry weights results before and after filtration, Tables S1 and S2: 1H-NMR average number molecular weight (Mn) and PEG repetitive units (n) present on samples of synthesized terpolymer combinations, Table S3: Wet weight of 100 µL samples of three different terpolymer batches before and after filtration using PES membrane, Table S4: Dry weight samples of terpolymer batches before and after filtration using PES membrane.

## References

[1] Lee J., Cuddihy M.J., Kotov N.A. (2008). Three-dimensional cell culture matrices: State of the art. Tissue Eng. Part B Rev..

[2] Sant S., Hancock M.J., Donnelly J.P., Iyer D., Khademhosseini A. (2010). Biomimetic gradient hydrogels for tissue engineering. Can. J. Chem. Eng..

[3] Lee K.Y., Mooney D.J. (2001). Hydrogels for tissue engineering. Chem. Rev..

[4] Haddock R., Lin-Gibson S., Lumelsky N., McFarland R., Roy K., Zhang J., McFarland C. Manufacturing Cell Therapies: The Paradigm Shift in Healthcare of this Century. NAM Perspect..

[5] Abdeen A.A., Saha K. (2017). Manufacturing Cell Therapies Using Engineered Biomaterials. Trends Biotechnol..

[6] del Río E.P., Miguel M.M., Veciana J., Ratera I., Guasch J. (2018). Artificial 3D Culture Systems for T Cell Expansion. ACS Omega.

[7] Peppas N.A., Bures P., Leobandung W., Ichikawa H. (2000). Hydrogels in pharmaceutical formulations. Eur. J. Pharm. Biopharm..

[8] Wang Y., Wang J., Yuan Z., Han H., Li T., Li L., Guo X. (2017). Chitosan cross-linked poly(acrylic acid) hydrogels: Drug release control and mechanism. Colloids Surfaces B Biointerfaces.

[9] Melo B.C., Paulino F.A.A., Cardoso V.A., Pereira A.G.B., Fajardo A.R., Rodrigues F.H.A. (2018). Cellulose nanowhiskers improve the methylene blue adsorption capacity of chitosan-g-poly(acrylic acid) hydrogel. Carbohydr. Polym..

[10] Qayyum A.S., Jain E., Kolar G., Kim Y., Sell S.A., Zustiak S.P. (2017). Design of electrohydrodynamic sprayed polyethylene glycol hydrogel microspheres for cell encapsulation. Biofabrication.

[11] Mora N.L., Gao Y., Gutierrez M.G., Peruzzi J., Bakker I., Peters R.J.R.W., Siewert B., Bonnet S., Kieltyka R.E., van Hest J.C.M. (2017). Evaluation of dextran(ethylene glycol) hydrogel films for giant unilamellar lipid vesicle production and their application for the encapsulation of polymersomes. Soft Matter..

[12] Barnett H.H., Heimbuck A.M., Pursell I., Hegab R.A., Sawyer B.J., Newman J.J., Caldorera-Moore M.E. (2019). Poly (ethylene glycol) hydrogel scaffolds with multiscale porosity for culture of human adipose-derived stem cells. J. Biomater. Sci. Polym. Ed..

[13] Hu W., Feng X., Liu X., Dai S., Zeng W., Jiang Q., Chen B., Quan C., Sun K., Zhang C. (2016). Poly(γ-glutamic acid) modulates the properties of poly(ethylene glycol) hydrogel for biomedical applications. J. Biomater. Sci. Polym. Ed..

[14] Kamoun E.A., Kenawy E.R.S., Chen X. (2017). A review on polymeric hydrogel membranes for wound dressing applications: PVA-based hydrogel dressings. J. Adv. Res..

[15] Rizwan M., Yao Y., Gorbet M.B., Tse J.W., Anderson D.E.J., Hinds M.T., Yim E.K.F. (2020). One-Pot Covalent Grafting of Gelatin on Poly(Vinyl Alcohol) Hydrogel to Enhance Endothelialization and Hemocompatibility for Synthetic Vascular Graft Applications. ACS Appl. Bio Mater..

[16] Hayes J.C., Curley C., Tierney P., Kennedy J.E. (2016). Biomechanical analysis of a salt-modified polyvinyl alcohol hydrogel for knee meniscus applications, including comparison with human donor samples. J. Mech. Behav. Biomed. Mater..

[17] Kapoor S., Kundu S.C. (2016). Silk protein-based hydrogels: Promising advanced materials for biomedical applications. Acta Biomater..

[18] Eslami M., Vrana N.E., Zorlutuna P., Sant S., Jung S., Masoumi N., Khavari-Nejad R.A., Javadi G., Khademhosseini A. (2014). Fiber-reinforced hydrogel scaffolds for heart valve tissue engineering. J. Biomater. Appl..

[19] Tseng H., Puperi D.S., Kim E.J., Ayoub S., Shah J.V., Cuchiara M.L., West J.L., Grande K.J. (2014). Anisotropic poly(ethylene glycol)/polycaprolactone (PEG/PCL) hydrogel-fiber composites for heart valve tissue engineering. Tissue Eng..

[20] Ravishankar P., Ozkizilcik A., Husain A., Balachandran K. (2021). Anisotropic Fiber-Reinforced Glycosaminoglycan Hydrogels for Heart Valve Tissue Engineering. Tissue Eng. Part A.

[21] Nilghaz A., Hoo S., Shen W., Lu X., Chan P.P.Y. (2018). Multilayer cell culture system supported by thread. Sens. Actuators B Chem..

[22] Choi A., Seo K.D., Yoon H., Han S.J., Kim D.S. (2019). Bulk poly(: N -isopropylacrylamide) (PNIPAAm) thermoresponsive cell culture platform: Toward a new horizon in cell sheet engineering. Biomater. Sci..

[23] Kobayashi J., Akiyama Y., Yamato M., Shimizu T., Okano T. (2018). Design of Temperature-Responsive Cell Culture Surfaces for Cell Sheet-Based Regenerative Therapy and 3D Tissue Fabrication. Adv. Exp. Med. Biol..

[24] Mellati A., Kiamahalleh M.V., Madani S.H., Dai S., Bi J., Jin B., Zhang H. (2016). Poly(N-isopropylacrylamide) hydrogel/chitosan scaffold hybrid for three-dimensional stem cell culture and cartilage tissue engineering. J. Biomed. Mater. Res. Part A.

[25] Higgins W., Kozlovskaya V., Alford A., Ankner J., Kharlampieva E. (2016). Stratified Temperature-Responsive Multilayer Hydrogels of Poly(N-vinylpyrrolidone) and Poly(N-vinylcaprolactam): Effect of Hydrogel Architecture on Properties. Macromolecules.

[26] Macková H., Plichta Z., Hlídková H., Sedláček O., Konefal R., Sadakbayeva Z., Dušková-Smrčková M., Horák D., Kubinová Š. (2017). Reductively Degradable Poly(2-hydroxyethyl methacrylate) Hydrogels with Oriented Porosity for Tissue Engineering Applications. ACS Appl. Mater. Interfaces.

[27] He H., Sofman M., Wang A.J.S., Ahrens C.C., Wang W., Griffith L.G., Hammond P.T. (2020). Engineering Helical Modular Polypeptide-Based Hydrogels as Synthetic Extracellular Matrices for Cell Culture. Biomacromolecules.

[28] Villa C., Martello F., Erratico S., Tocchio A., Belicchi M., Lenardi C., Torrente Y. (2017). P(NIPAAM–co-HEMA) thermoresponsive hydrogels: An alternative approach for muscle cell sheet engineering. J. Tissue Eng. Regen. Med..

[29] Chang C.W., Nguyen T.H., Maynard H.D. (2010). Thermoprecipitation of glutathione S-transferase by glutathione- poly(Nisopropylacrylamide) prepared by RAFT polymerization. Macromol. Rapid Commun..

[30] Raj G.L.T. (2017). Hydrogels.

[31] Cruz-Acuña R., García A.J. (2017). Synthetic hydrogels mimicking basement membrane matrices to promote cell-matrix interactions. Matrix Biol..

[32] Tsuda Y., Shimizu T., Yamato M., Kikuchi A., Sasagawa T., Sekiya S., Kobayashi J., Chen G., Okano T. (2007). Cellular control of tissue architectures using a three-dimensional tissue fabrication technique. Biomaterials.

[33] Yang N., Zhou K. (2014). Effective method for multi-scale gradient porous scaffold design and fabrication. Mater. Sci. Eng. C.

[34] Hu Y., Grainger D.W., Winn S.R., Hollinger J.O. (2002). Fabrication of poly (α-hydroxy acid) foam scaffolds using multiple solvent systems. J. Biomed. Mater. Res..

[35] Levine B.L., Miskin J., Wonnacott K., Keir C. (2017). Global Manufacturing of CAR T-Cell Therapy. Mol. Ther. Methods Clin. Dev..

[36] Ward M.A., Georgiou T.K. (2011). Thermoresponsive polymers for biomedical applications. Polymers.

[37] Klouda L. (2015). Thermoresponsive hydrogels in biomedical applications A seven-year update. Eur. J. Pharm. Biopharm..

[38] Zhang Q., Weber C., Schubert U.S., Hoogenboom R. (2017). Thermoresponsive polymers with lower critical solution temperature: From fundamental aspects and measuring techniques to recommended turbidimetry conditions. Mater. Horiz..

[39] Hacker M.C., Klouda L., Ma B.B., Kretlow J.D., Mikos A.G. (2008). Synthesis and characterization of injectable, thermally and chemically gelable, amphiphilic poly(N-isopropylacrylamide)-based macromers. Biomacromolecules.

[40] Piest M. (2011). Boronic Acid Functionalized Polymers and Hydrogels for Biomedical Applications.

[41] Bull S.D., Davidson M.G., van den Elsen J.M.H., Fossey J.S., Jenkins A.T.A., Jiang Y.B., Kubo Y., Marken F., Sakurai K., Zhao J. (2013). Exploiting the reversible covalent bonding of boronic acids: Recognition, sensing, and assembly. Acc. Chem. Res..

[42] Ma W.M.J., Morais M.P.P., D’Hooge F., van den Elsen J.M.H., Cox J.P.L., James T.D., Fossey J.S. (2009). Dye displacement assay for saccharide detection with boronate hydrogels. Chem. Commun..

[43] Alexander A., Ajazuddin, Khan J., Saraf S., Saraf S. (2014). Polyethylene glycol (PEG)-Poly(N-isopropylacrylamide) (PNIPAAm) based thermosensitive injectable hydrogels for biomedical applications. Eur. J. Pharm. Biopharm..

[44] Peppas N.A., Keys K.B., Torres-Lugo M., Lowman A.M. (1999). Poly(ethylene glycol)-containing hydrogels in drug delivery. J. Control. Release.

[45] Akimoto A.M., Hasuike E., Tada H., Nagase K., Okano T., Kanazawa H., Yoshida R. (2016). Design of Tetra-arm PEG-crosslinked Thermoresponsive Hydrogel for 3D Cell Culture. Anal. Sci..

[46] Akimoto A.M., Niitsu E.H., Nagase K., Okano T., Kanazawa H., Yoshida R. (2018). Mesenchylmal stem cell culture on poly(N-isopropylacrylamide) hydrogel with repeated thermo-stimulation. Int. J. Mol. Sci..

[47] Cui X., Hartanto Y., Wu C., Bi J., Dai S., Zhang H. (2018). Tuning microenvironment for multicellular spheroid formation in thermo-responsive anionic microgel scaffolds. J. Biomed. Mater. Res. Part A.

[48] Konishi T., Akimoto A.M., Nishimoto T., Tokura Y., Tenjimbayashi M., Homma K., Matsukawa K., Kaku T., Hiruta Y., Nagase K. (2019). Crosslinked Poly(N-Isopropylacrylamide)-Based Microfibers as Cell Manipulation Materials with Prompt Cell Detachment. Macromol. Rapid Commun..

[49] Oroojalian F., Jahanafrooz Z., Chogan F., Rezayan A.H., Malekzade E., Rezaei S.J.T., Nabid M.R., Sahebkar A. (2019). Synthesis and evaluation of injectable thermosensitive penta-block copolymer hydrogel (PNIPAAm-PCL-PEG-PCL-PNIPAAm) and star-shaped poly(CL–CO–LA)-b-PEG for wound healing applications. J. Cell. Biochem..

[50] Reddy R.M., Srivastava A., Kumar A. (2013). Monosaccharide-Responsive Phenylboronate-Polyol Cell Scaffolds for Cell Sheet and Tissue Engineering Applications. PLoS ONE.

[51] Shakya A.K., Holmdahl R., Nandakumar K.S., Kumar A. (2013). Characterization of chemically defined poly-N-isopropylacrylamide based copolymeric adjuvants. Vaccine.

[52] Ao W., Han J., Wang L., Li C., Mao Y., Wang Y. (2019). Novel Fractional Purification Approach of Crude Polysaccharides via Boronic Acid-Tagged Thermoresponsive Triblock Copolymers. ACS Sustain. Chem. Eng..

[53] Sun W., Dai R., Li B., Dai G., Wang D., Yang D., Chu P., Deng Y., Luo A. (2019). Combination of three functionalized temperature-sensitive chromatographic materials for serum protein analysis. Molecules.

[54] Uǧuzdoǧan E., Denkbaş E.B., Tuncel A. (2002). RNA-sensitive N-isopropylacrylamide/vinylphenylboronic acid random copolymer. Macromol. Biosci..

[55] Izunobi J.U., Higginbotham C.L. (2011). Polymer molecular weight analysis by 1H NMR spectroscopy. J. Chem. Educ..

[56] Çimen E.K., Rzaev Z.M.O., Pişkin E. (2005). Bioengineering functional copolymers: V. Synthesis, LCST, and thermal behavior of poly(N-isopropyl acrylamide-co-p-vinylphenylboronic acid). J. Appl. Polym. Sci..

[57] Pollock J.F., Healy K.E. (2010). Mechanical and swelling characterization of poly(N-isopropyl acrylamide -co- methoxy poly(ethylene glycol) methacrylate) sol-gels. Acta Biomater..

[58] Castilla-Casadiego D.A., Ramos-Avilez H.V., Herrera-Posada S., Calcagno B., Loyo L., Shipmon J., Acevedo A., Quintana A., Almodovar J. (2016). Engineering of a Stable Collagen Nanofibrous Scaffold with Tunable Fiber Diameter, Alignment, and Mechanical Properties. Macromol. Mater. Eng..

[59] Court K.A., Hatakeyama H., Wu S.Y., Lingegowda M.S., Rodríguez-Aguayo C., López-Berestein G., Ju-Seog L., Rinaldi C., Juan E.J., Sood A.K. (2017). HSP70 Inhibition Synergistically Enhances the Effects of Magnetic Fluid Hyperthermia in Ovarian Cancer. Mol. Cancer Ther..

[60] Lian X., Zhang J., Azarin S.M., Zhu K., Hazeltine L.B., Bao X., Hsiao C., Kamp T.J., Palecek S.P. (2013). Directed cardiomyocyte differentiation from human pluripotent stem cells by modulating Wnt/β-catenin signaling under fully defined conditions. Nat. Protoc..

[61] (2005). Invitrogen, LIVE/DEAD Viability/Cytotoxicity Kit for Mammalian Cells. Prod. Information. Cat. Number MP 03224. https://assets.fishersci.com/TFS-Assets/LSG/manuals/mp03224.pdf.

[62] Pinzon-Herrera L., Mendez-Vega J., Mulero-Russe A., Castilla-Casadiego D.A., Almodovar J. (2020). Real-time monitoring of human Schwann cells on heparin-collagen coatings reveals enhanced adhesion and growth factor response. J. Mater. Chem. B..

[63] Türk M., Rzayev Z.M.O., Kurucu G. (2010). Bioengineering functional copolymers. XII. Interaction of boron-containing and PEO branched derivatives of poly(MA-alt-MVE) with HeLa cells. Health.

[64] Kahraman G., Beşkardeş O., Rzaev Z.M.O., Pişkin E. (2004). Bioengineering polyfunctional copolymers. VII. Synthesis and characterization of copolymers of p-vinylphenyl boronic acid with maleic and citraconic anhydrides and their self-assembled macrobranched supramolecular architectures. Polymer.

[65] Ye Y., Huang J., Wang X. (2015). Fabrication of a Self-Cleaning Surface via the Thermosensitive Copolymer Brush of P(NIPAAm-PEGMA). ACS Appl. Mater. Interfaces.

[66] Zhang X.Z., Yang Y.Y., Chung T.S., Ma K.X. (2001). Preparation and characterization of fast response macroporous poly(N-isopropylacrylamide) hydrogels. Langmuir.

[67] Luzon M., Boyer C., Peinado C., Corrales T., Whittaker M., Tao L., Davis T.P. (2010). Water-soluble, thermoresponsive, hyperbranched copolymers based on PEG-methacrylates: Synthesis, characterization, and LCST behavior. J. Polym. Sci. Part A Polym. Chem..

[68] Feng Y., Zhao H., Behl M., Lendlein A., Guo J., Yang D. (2013). Grafting of poly(ethylene glycol) monoacrylates on polycarbonateurethane by UV initiated polymerization for improving hemocompatibility. J. Mater. Sci. Mater. Med..

[69] Amjadi S., Hamishehkar H., Ghorbani M. (2019). A novel smart PEGylated gelatin nanoparticle for co-delivery of doxorubicin and betanin: A strategy for enhancing the therapeutic efficacy of chemotherapy. Mater. Sci. Eng. C.

[70] Rzayev Z.M.O., Beşkardeş O. (2007). Boron-Containing Functional Copolymers for Bioengineering Applications. Collect. Czechoslov. Chem. Commun..

[71] Marsili L., Bo M.D., Eisele G., Donati I., Berti F., Toffoli G. (2021). Characterization of thermoresponsive poly-n-vinylcaprolactam polymers for biological applications. Polymers.

[72] Michel E., Filali M., Aznar R., Porte G., Appell J. (2000). Percolation in a model transient network: Rheology and dynamic light scattering. Langmuir.

[73] Kanao M., Matsuda Y., Sato T. (2003). Characterization of polymer solutions containing a small amount of aggregates by static and dynamic light scattering. Macromolecules.

[74] Burchard W., Richtering W. (2007). Dynamic light scattering from polymer solutions. Relax. Polym..

[75] Norisuye T., Morinaga T., Tran-Cong-Miyata Q., Goto A., Fukuda T., Shibayama M. (2005). Comparison of the gelation dynamics for polystyrenes prepared by conventional and living radical polymerizations: A time-resolved dynamic light scattering study. Polymer.

[76] García-Peñas A., Biswas C.S., Liang W., Wang Y., Yang P., Stadler F.J. (2019). Effect of hydrophobic interactions on lower critical solution temperature for poly(N-isopropylacrylamide-co-dopamine methacrylamide) copolymers. Polymers.

[77] Luan B., Muir B.W., Zhu J., Hao X. (2016). A RAFT copolymerization of NIPAM and HPMA and evaluation of thermo-responsive properties of poly(NIPAM-co-HPMA). RSC Adv..

[78] Ghasem N.M., Al-Marzouqi M.H. (2011). Effects of shear rate, temperature, and polymer composition on the shear stress of polyethersulfone/1-methyl-2-pyrrolidone cast solutions. J. Chem. Eng. Data.

[79] Torres J.M., Stafford C.M., Vogt B.D. (2009). Elastic modulus of amorphous polymer thin films: Relationship to the glass transition temperature. ACS Nano.

[80] Rahimzadeh A., Rutsch M., Kupnik M., von Klitzing R. (2021). Visualization of Acoustic Energy Absorption in Confined Aqueous Solutions by PNIPAM Microgels: Effects of Bulk Viscosity. Langmuir.

[81] Otsuka H., Nagasaki Y., Kataoka K. (2004). Characterization of aldehyde-PEG tethered surfaces: Influence of PEG chain length on the specific biorecognition. Langmuir.

[82] Jiao T., Clifton R.J., Converse G.L., Hopkins R.A. (2012). Measurements of the effects of decellularization on viscoelastic properties of tissues in ovine, baboon, and human heart valves. Tissue Eng. Part A.

[83] Guimarães C.F., Gasperini L., Marques A.P., Reis R.L. (2020). The stiffness of living tissues and its implications for tissue engineering. Nat. Rev. Mater..

[84] Jacot J.G., Martin J.C., Hunt D.L. (2010). Mechanobiology of cardiomyocyte development. J. Biomech..

[85] Hickey J.W., Dong Y., Chung J.W., Salathe S.F., Pruitt H.C., Li X., Chang C., Fraser A.K., Bessell C.A., Ewald A.J. (2019). Engineering an Artificial T-Cell Stimulating Matrix for Immunotherapy. Adv. Mater..

[86] Mao A.S., Shin J.W., Mooney D.J. (2016). Effects of substrate stiffness and cell-cell contact on mesenchymal stem cell differentiation. Biomaterials.

[87] Rigon R.B., Gonçalez M.L., Severino P., Alves D.A., Santana M.H.A., Souto E.B., Chorilli M. (2018). Solid lipid nanoparticles optimized by 22 factorial design for skin administration: Cytotoxicity in NIH3T3 fibroblasts. Colloids Surfaces B Biointerfaces.

[88] Abràmoff M.D., Magalhães P.J., Ram S.J. (2004). Image processing with imageJ. Biophotonics Int..

[89] Han S.J., Kwon S., Kim K.S. (2021). Challenges of applying multicellular tumor spheroids in preclinical phase. Cancer Cell Int..

[90] Knight E., Przyborski S. (2015). Advances in 3D cell culture technologies enabling tissue-like structures to be created in vitro. J. Anat..

[91] Loessner D., Stok K.S., Lutolf M.P., Hutmacher D.W., Clements J.A., Rizzi S.C. (2010). Bioengineered 3D platform to explore cell-ECM interactions and drug resistance of epithelial ovarian cancer cells. Biomaterials.

[92] Guo Z., Zhang T., Fang K., Liu P., Li M., Gu N. (2016). The effect of porosity and stiffness of glutaraldehyde cross-linked egg white scaffold simulating aged extracellular matrix on distribution and aggregation of ovarian cancer cells. Colloids Surfaces A Physicochem. Eng. Asp..

[93] Maldonado M., Wong L.Y., Echeverria C., Ico G., Low K., Fujimoto T., Johnson J.K., Nam J. (2015). The effects of electrospun substrate-mediated cell colony morphology on the self-renewal of human induced pluripotent stem cells. Biomaterials.

[94] Lou Y.R., Kanninen L., Kaehr B., Townson J.L., Niklander J., Harjumäki R., Brinker C.J., Yliperttula M. (2015). Silica bioreplication preserves three-dimensional spheroid structures of human pluripotent stem cells and HepG2 cells. Sci. Rep..

[95] Tong Z., Solanki A., Hamilos A., Levy O., Wen K., Yin X., Karp J.M. (2015). Application of biomaterials to advance induced pluripotent stem cell research and therapy. EMBO J..

[96] Tomaskovic-Crook E., Gu Q., Rahim S.N.A., Wallace G.G., Crook J.M. (2020). Conducting Polymer Mediated Electrical Stimulation Induces Multilineage Differentiation with Robust Neuronal Fate Determination of Human Induced Pluripotent Stem Cells. Cells.

