# Supplementary material for: Synthetic Thermo-Responsive Terpolymers as Tunable Scaffolds for Cell Culture Applications
Source: Polymers (Basel). 2022 Oct 17;14(20):4379. doi: 10.3390/polym14204379 (PMC9611013; doi:10.3390/polym14204379)
Supplement: Supplementary file 1 [file polymers-14-04379-s001.zip › polymers-1924637-supplementary.pdf]

1. Terpolymer Characterization

Table S1: ^{1}H-NMR average number molecular weight (Mn) and PEG repetitive units (n) present on samples of synthesized P400 terpolymer combinations.

Reactors	2:3:95_P400	2:4:94_P400	4:4:92_P400	4:8:88_P400	4:12:84_P400
R1	4013.10	2646.35	2788.86	1592.84	1194.07
R2	3255.51	2854.65	2637.21	1648.25	1223.93
R3	3485.35	2751.00	2595.88	1633.90	1243.60
R4	2468.33	1873.39	3335.88	1757.02	1231.40
R5	3543.34	2854.01	2821.58	1752.35	1217.57
R6	NA	NA	NA	1584.13	1252.35
**Average**	**3353.12**	**2595.88**	**2835.88**	**1661.42**	**1233.77**
**STDEV**	**566.04**	**412.97**	**295.59**	**76.17**	**14.20**
**n**	**5**	**5**	**5**	**6**	**6**
**SEM**	**253.14**	**184.69**	**132.19**	**31.10**	**5.80**

Table S2: ^{1}H-NMR average number molecular weight (Mn) and PEG repetitive units (n) present on samples of synthesized P1000 terpolymer combinations.

Reactors	2:3:95_P1000	2:4:94_P1000	4:4:92_P1000	10:4:86_P1000
R1	4955.04	2014.86	3285.76	2591.46
R2	3860.56	3452.76	3185.85	2608.15
R3	3849.79	3157.25	3755.97	2682.78
R4	3724.10	3377.70	3157.83	2599.30
R5	4105.13	2594.90	3196.88	2515.46
R6	3868.65	3370.99	1855.69	3141.09
**Average**	**4060.55**	**3125.21**	**3316.46**	**2689.71**
**STDEV**	**455.31**	**368.06**	**250.32**	**227.44**
**n**	**6**	**6**	**6**	**6**
**SEM**	**185.88**	**150.26**	**102.19**	**92.85**

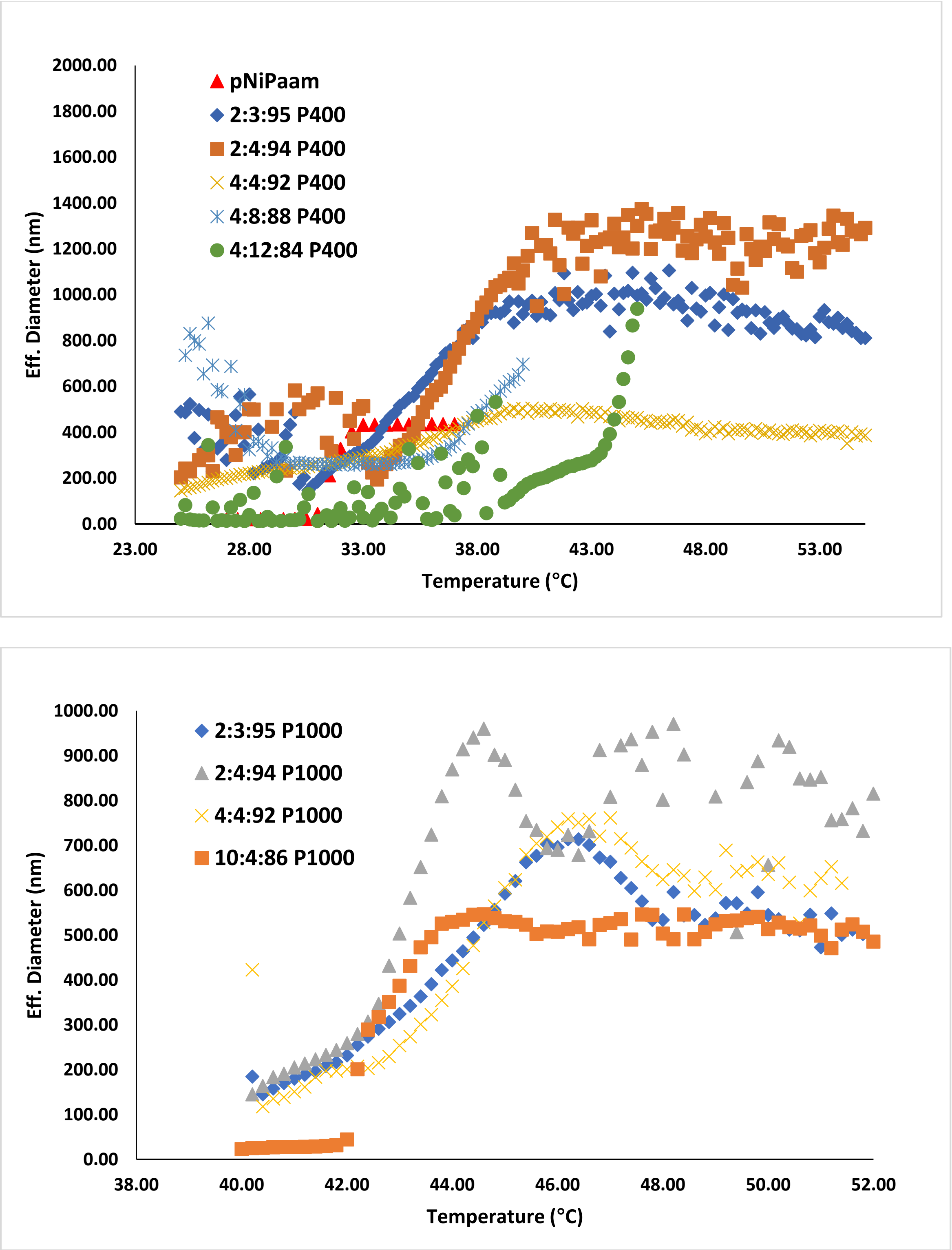

Figure S1: Effect of temperature on effective diameter of pNipAAM as baseline and terpolymer with PEGMMA400 and PEGMMA1000 in synthesis.

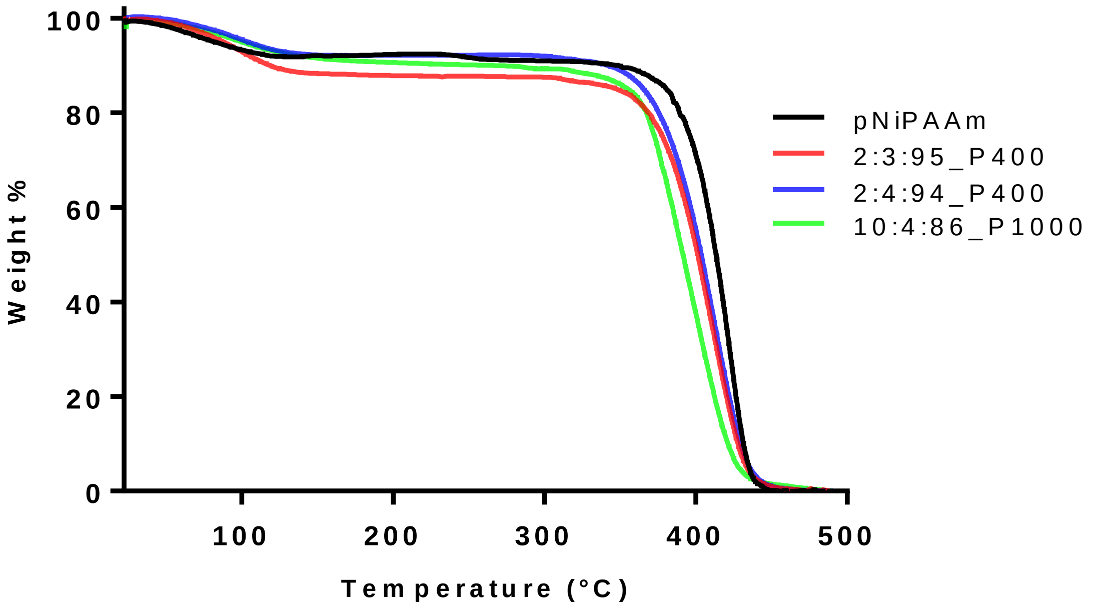

Figure S2: Thermal gravimetric analysis for pNiPAAm pure, 2:3:95_P400, 2:4:94_P400 and 10:4:86_P1000 terpolymer combinations.

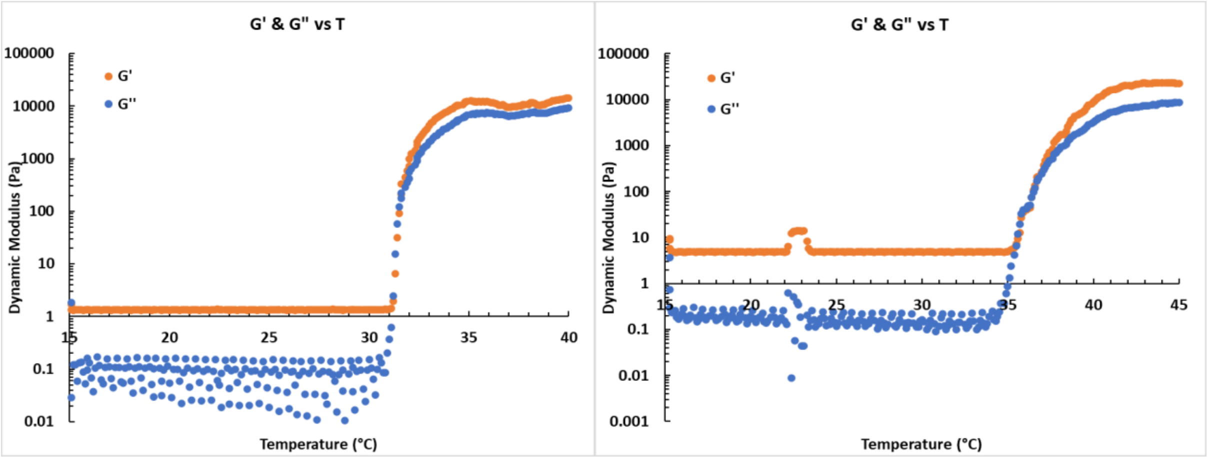

Figure S3: Dynamic modulus in function of temperature for pNipAAm (left) and 2:3:95_P400 (right) at 15wt%.

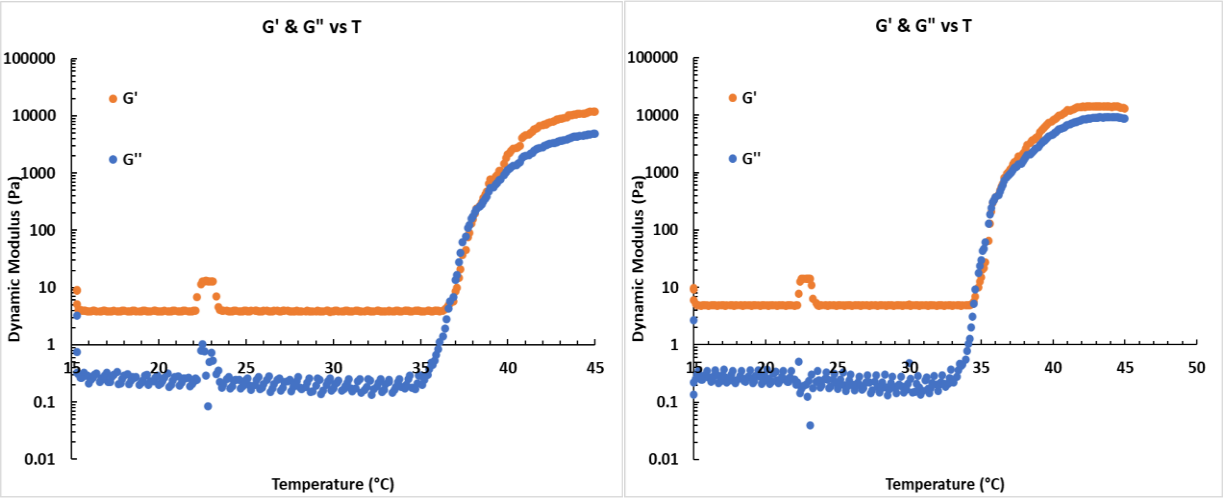

Figure S4: Dynamic modulus in function of temperature for 2:4:94_P400 (left) and 4:4:92_P400 (right) at 15wt%.

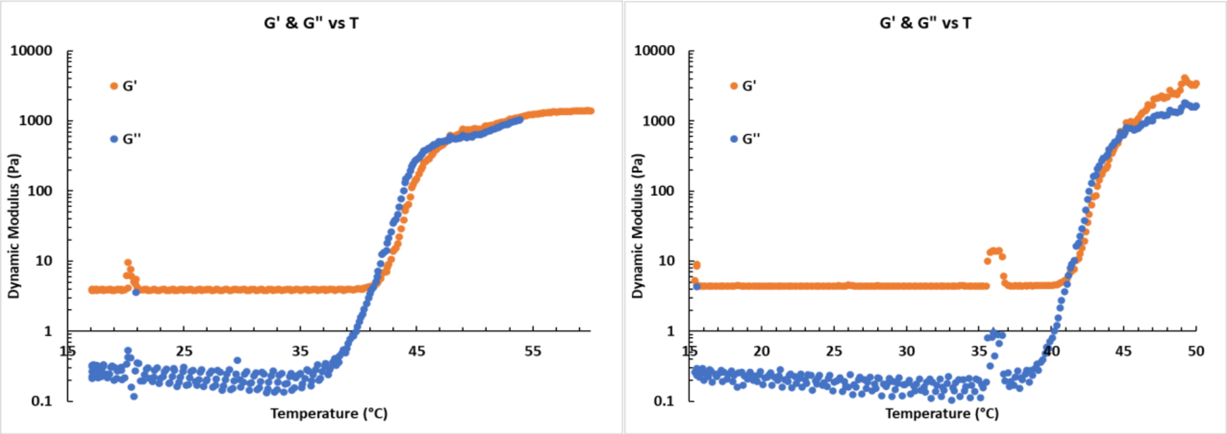

Figure S5: Dynamic modulus in function of temperature for 4:8:88_P400 (left) and 4:12:84_P400 (right) at 15wt%.

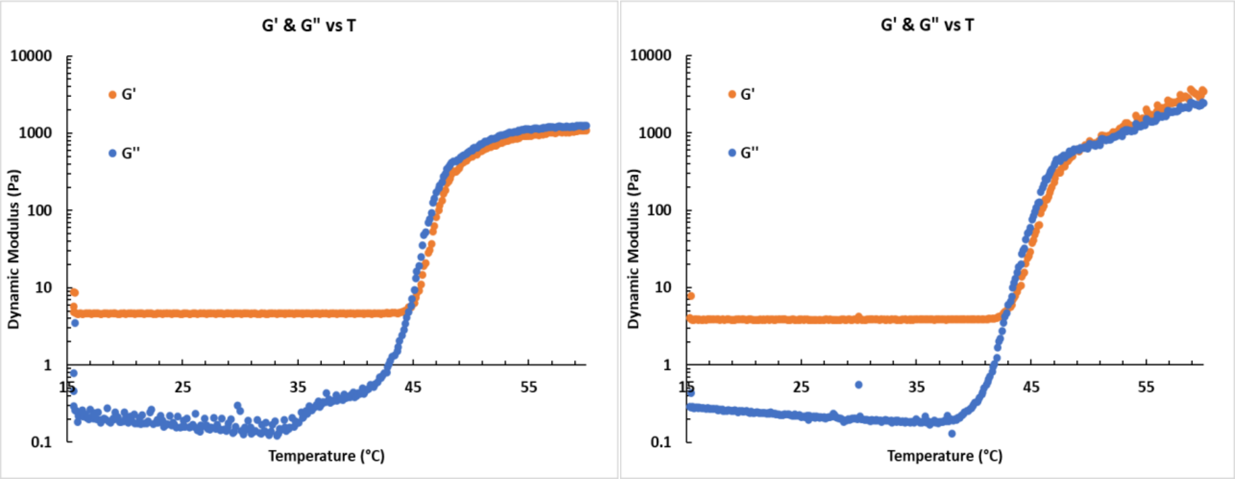

Figure S6: Dynamic modulus in function of temperature for 2:3:95_P1000 (left) and 2:4:94_P1000 (right) at 15wt%.

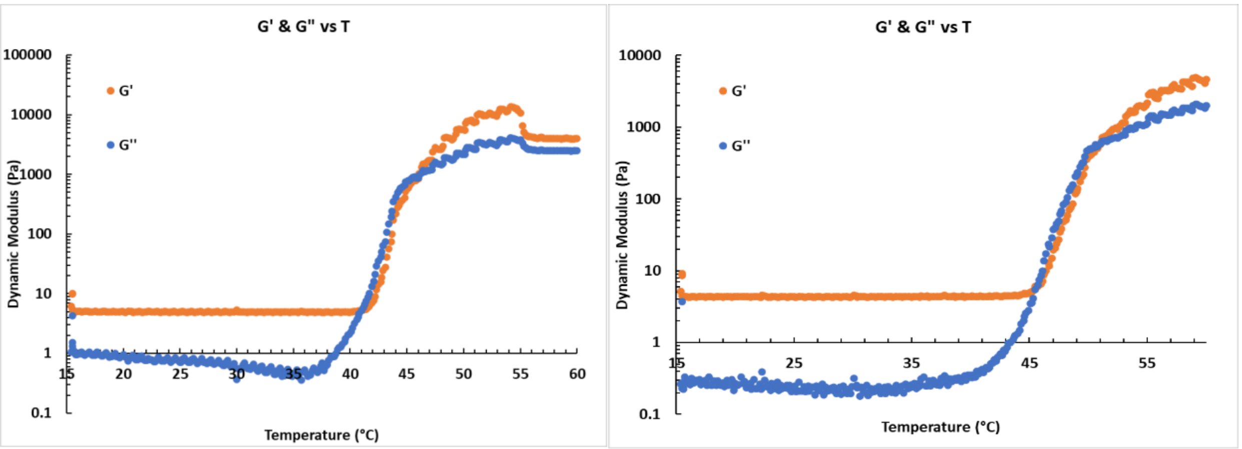

Figure S7: Dynamic modulus in function of temperature for 4:4:92_P1000 (left) and 10:4:86_P1000 (right) at 15wt%.

2. Cell culture

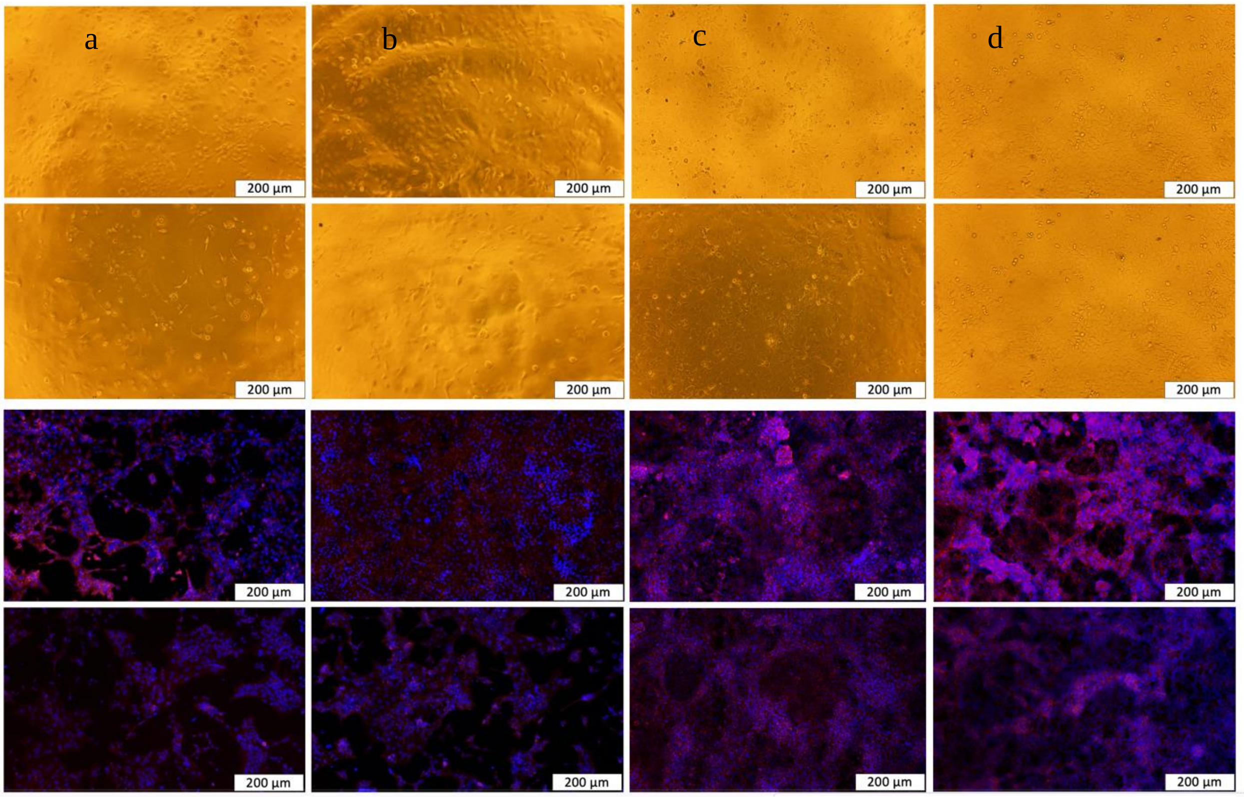

Figure S8. Mixing condition used to encapsulate SKOV-3 ovarian cancer cell line within terpolymer scaffolds. Cells appeared to fall to the bottom of the well before gelation was obtained, resulting in a 2D growth on the well surface. Rows show cells pictures inside 2:3:95_P400 (a), 2:4:92_P400 (b), 10:4:86_P1000 terpolymers scaffolds and the control (d) without terpolymer. Pictures with yellow background were taken on day 4 of incubation, while the merged fluorescence pictures in day 10 of incubation. In both cases, pictures on the top and bottom columns represent

the scaffolds of 15 wt.% and 20 wt.%, respectively. The blue color represents the cell's nucleus, and the red color the cell's cytoskeleton.

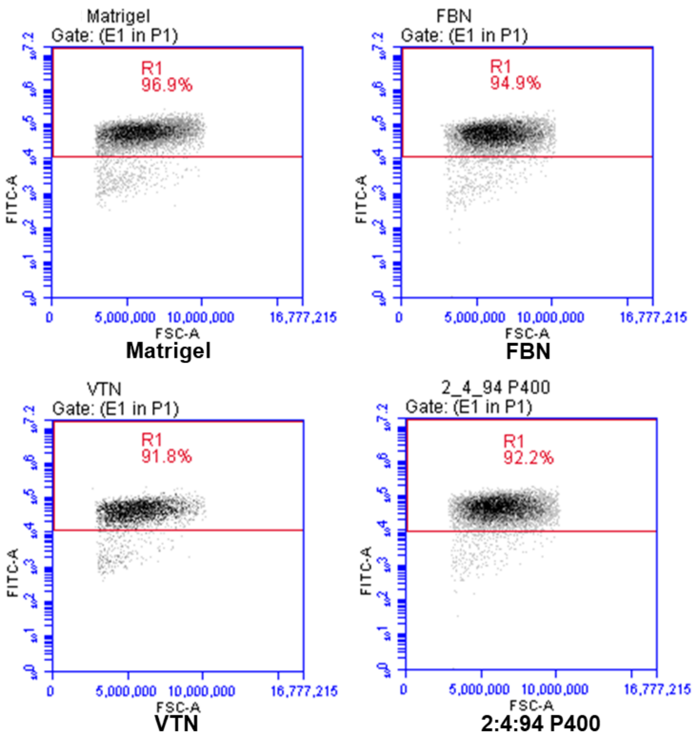

Figure S9. Pluripotency plots for Matrigel, Fibronectin (FBN), Vitronectin (VTN), and 2:4:94 P400 terpolymer combination.

3. Statistical analysis

3.1. Filtration effects in terpolymer solution.

Three different batches, different days of synthesis, and random reactors, of 2:4:94_P400 terpolymer combination, were used in this experiment. A solution of 40wt.% terpolymer in D.I water of each selected batch was prepared to be filtrated, and by differences in weight, evaluate the effect of the PES membrane used. A sample of 100 µL was weight in triplicate before and after filtration to observe any effect by weight in the process. The results of samples weight, wet and dry (after the weekend), are shown in Tables 1 and 2, respectively.

Results were analyzed statistically in the Minitab program using a 2 sample T-Test analysis to find any significant difference in the results of the means before and after the filtration process by family batches. The null hypothesis of the T-Test indicated that the population means evaluated of the same family are the same, 06/20/2019_R6 (R6), 07/22/2020_composite (C), and 08/19/2020_R4 (R4). In contrast, the alternative hypothesis affirms population means are different.

Table S3: Wet weight of 100 µL samples of three different terpolymer batches, 06/20/2019_R6 (R6), 07/22/2020_composite (C), and 08/19/2020_R4 (R4), before and after filtration using PES membrane.

Sample ID	R6-A	R6-D	C-A	C-D	R4-A	R4-D
**Replicate 1**	0.08030	0.08220	0.07610	0.07690	0.08050	0.08690
**Replicate 2**	0.08020	0.07770	0.07760	0.07700	0.07730	0.07950
**Replicate 3**	0.07940	0.08410	0.07510	0.07870	0.08250	0.07740
**Average**	0.07997	0.08133	0.07627	0.07753	0.08010	0.08127
**STDEV**	0.00049	0.00329	0.00126	0.00101	0.00262	0.00499

Table S4: Dry weight of 100 µL samples of three different terpolymer batches, 06/20/2019_R6 (R6), 07/22/2020_composite (C), and 08/19/2020_R4 (R4), before and after filtration using PES membrane.

Sample ID	R6-A_dry	R6-D_dry	C-A_dry	C-D_dry	R4-A_dry	R4-D_dry
**Replicate 1**	0.05080	0.05340	0.04710	0.04780	0.05080	0.05220
**Replicate 2**	0.05200	0.05030	0.04770	0.04830	0.04920	0.05060
**Replicate 3**	0.05090	0.05420	0.04690	0.04900	0.05180	0.04790
**Average**	0.05123	0.05263	0.04723	0.04837	0.05060	0.05023
**STDEV**	0.00067	0.00206	0.00042	0.00060	0.00131	0.00217

Since, in all cases, evaluate before and after filtration with wet and dry samples, the p-value is higher than 0.05, there is not enough evidence to reject the null hypothesis. For this reason, with 95% significance, we can affirm that the means of the study population are statistically equivalent. The T-Test results, including the Boxplot, are shown following:

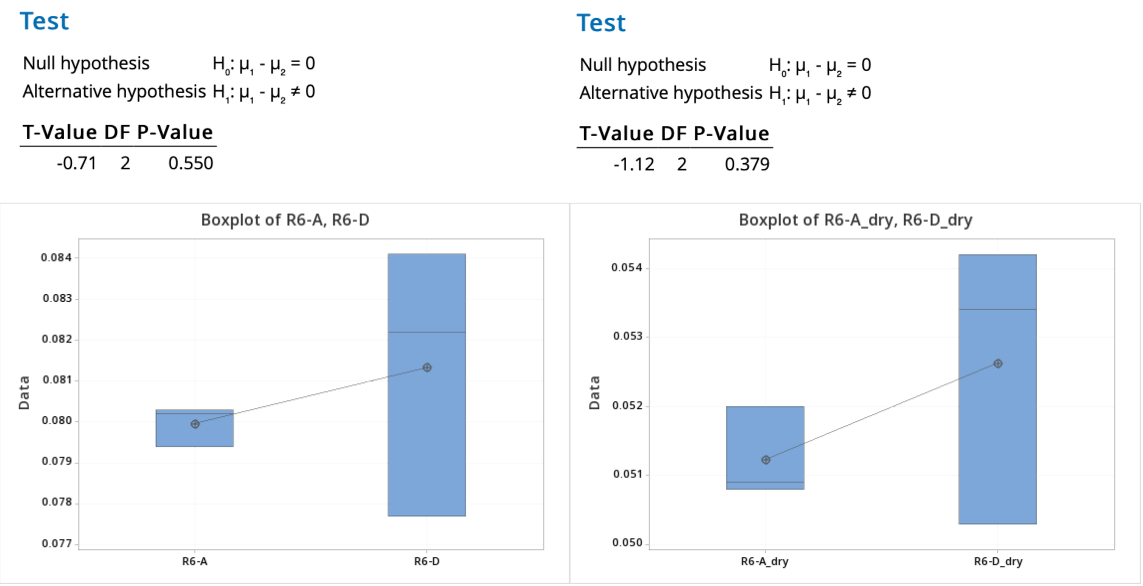

Figure S10: T-Test analysis in Minitab software of wet and dry weights results before and after filtration using the 06/20/2019_R6 (R6) sample.

Test

Null hypothesis $H_0: \mu_1 - \mu_2 = 0$

Alternative hypothesis $H_1: \mu_1 - \mu_2 \neq 0$

T-Value	DF	P-Value
-1.36	3	0.267

Test

Null hypothesis $H_0: \mu_1 - \mu_2 = 0$

Alternative hypothesis $H_1: \mu_1 - \mu_2 \neq 0$

T-Value	DF	P-Value
-2.68	3	0.075

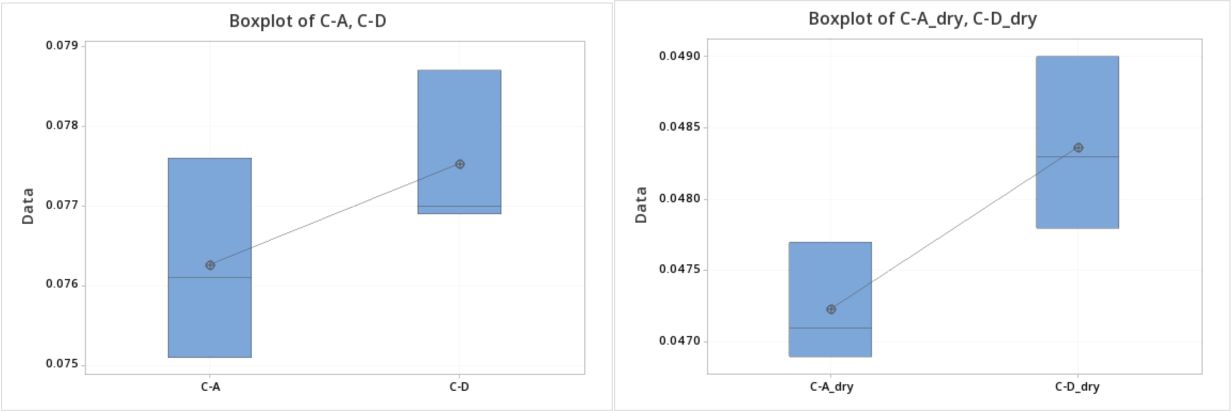

Figure S11: T-Test analysis in Minitab software of wet and dry weights results before and after filtration using a 07/22/2020_composite (C) sample.

Test

Null hypothesis $H_0: \mu_1 - \mu_2 = 0$

Alternative hypothesis $H_1: \mu_1 - \mu_2 \neq 0$

T-Value	DF	P-Value
-0.36	3	0.744

Test

Null hypothesis $H_0: \mu_1 - \mu_2 = 0$

Alternative hypothesis $H_1: \mu_1 - \mu_2 \neq 0$

T-Value	DF	P-Value
0.25	3	0.819

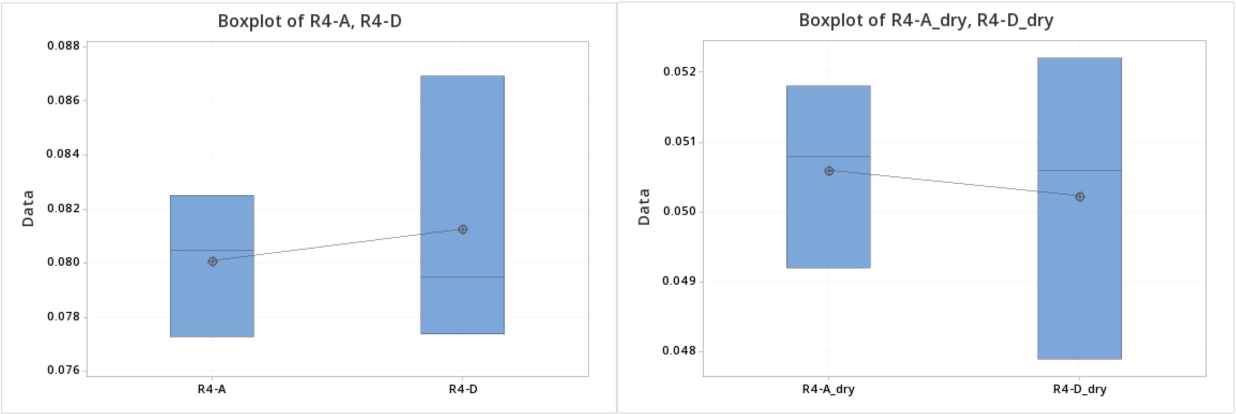

Figure S12: T-Test analysis in Minitab software of wet and dry weights results before and after filtration using the 08/19/2020_R4 (R4) sample.

3.2. H-NMR data

3.2.1. Number average Molecular weight (Mn)

Molecular weight P400 terpolymers

Tukey's multiple comparisons test	Mean Diff.	95.00% CI of diff.	Summary	Adjusted P Value
2:3:95_P400 vs. 2:4:94_P400	757.2	144.7 to 1370	*	0.0106
2:3:95_P400 vs. 4:4:92_P400	517.2	-95.32 to 1130	ns	0.1257
2:3:95_P400 vs. 4:8:88_P400	1692	1105 to 2278	****	<0.0001
2:3:95_P400 vs. 4:12:84_P400	2126	1539 to 2712	****	<0.0001
2:4:94_P400 vs. 4:4:92_P400	-240.0	-852.6 to 372.6	ns	0.7719
2:4:94_P400 vs. 4:8:88_P400	934.5	348.0 to 1521	***	0.0009
2:4:94_P400 vs. 4:12:84_P400	1369	782.2 to 1955	****	<0.0001
4:4:92_P400 vs. 4:8:88_P400	1174	588.0 to 1761	****	<0.0001
4:4:92_P400 vs. 4:12:84_P400	1609	1022 to 2195	****	<0.0001
4:8:88_P400 vs. 4:12:84_P400	434.3	-124.9 to 993.5	ns	0.1811

Molecular weight P1000 terpolymers

Tukey's multiple comparisons test	Mean Diff.	95.00% CI of diff.	Summary	Adjusted P Value
2:3:95_P1000 vs. 2:4:94_P1000	1066	260.4 to 1871	**	0.0071
2:3:95_P1000 vs. 4:4:92_P1000	987.5	182.1 to 1793	*	0.0129
2:3:95_P1000 vs. 10:4:86_P1000	1371	565.4 to 2176	***	0.0006
2:4:94_P1000 vs. 4:4:92_P1000	-78.25	-883.7 to 727.2	ns	0.9927
2:4:94_P1000 vs. 10:4:86_P1000	305.0	-500.4 to 1110	ns	0.7169
4:4:92_P1000 vs. 10:4:86_P1000	383.3	-422.1 to 1189	ns	0.5543

GP: 0.1234 (ns), 0.0332 (*), 0.0021 (**), 0.0002 (***), <0.0001 (****)

Outliers P400 terpolymers

Method<br>Grubbs (Alpha = 0.05)	2:3:95_P400	2:4:94_P400	4:4:92_P400	4:8:88_P400	4:12:84_P400
Number of points<br># Y values analyzed	5	5	5	6	6
Outliers	0	1	0	0	0
		R4			

Method:<br>ROUT (Q = 1%)	2:3:95_P400	2:4:94_P400	4:4:92_P400	4:8:88_P400	4:12:84_P400
Number of points<br># Y values analyzed	5	5	5	6	6
Outliers	0	0	0	0	0

Outliers P1000 terpolymers

Method<br>Grubbs (Alpha = 0.05)	2:3:95_P1000	2:4:94_P1000	4:4:92_P1000	10:4:86_P1000
Number of points<br># Y values analyzed	6	6	6	6
Outliers	1	0	1	1
	R1		R6	R6

Method:<br>ROUT (Q = 1%)	2:3:95_P1000	2:4:94_P1000	4:4:92_P1000	10:4:86_P1000
Number of points<br># Y values analyzed	6	6	6	6
Outliers	0	0	0	0

3.2.2. Molar composition

Outliers P400 terpolymers with the actual value

Method<br>Grubbs (Alpha = 0.05)	NiPAA<br>2:3:95	NiPAA<br>4:4:92	NiPAA<br>4:8:88	4-VPBA<br>4:8:88	P400<br>4:8:88	NiPAA<br>4:12:84	4-VPBA<br>4:12:84	P400<br>4:12:84
Number of points<br># Y values analyzed	6	6	7	7	7	7	7	7
Outliers	1	1	1	1	1	1	1	1
	R4	Actual	Actual	Actual	Actual	Actual	Actual	Actual

Method:<br>ROUT (Q = 1%)	NiPAA<br>4:4:92	NiPAA<br>4:8:88	P400<br>4:8:88	NiPAA<br>4:12:84	P400<br>4:12:84
Number of points<br># Y values analyzed	6	7	7	7	7
Outliers	1	1	1	1	1
	Actual	Actual	Actual	Actual	Actual

Outliers P1000 terpolymers with the actual value

Method<br>Grubbs (Alpha = 0.05)	P1000<br>2:4:94	NiPAA<br>4:4:92	4-VPBA<br>4:4:92	NiPAA<br>10:4:86	4-VPBA<br>10:4:86	P1000<br>10:4:84
Number of points<br># Y values analyzed	7	7	7	7	7	7
Outliers	1	1	1	1	1	1
	R3	Actual	Actual	Actual	Actual	Actual

Method:<br>ROUT (Q = 1%)	P1000<br>2:4:94	NiPAA<br>4:4:92	4-VPBA<br>4:4:92	NiPAA<br>10:4:86	4-VPBA<br>10:4:86

Number of points<br># Y values analyzed	7	7	7	7	7
Outliers	1	1	1	1	1
	R3	Actual	Actual	Actual	Actual
